# Palladium-catalyzed enantioselective three-component synthesis of α-arylglycine derivatives from glyoxylic acid, sulfonamides and aryltrifluoroborates

**DOI:** 10.3762/bjoc.19.52

**Published:** 2023-05-25

**Authors:** Bastian Jakob, Nico Schneider, Luca Gengenbach, Georg Manolikakes

**Affiliations:** 1 Department of Chemistry, RPTU Kaiserslautern-Landau, Erwin-Schrödinger-Str. Geb. 54, D-67663 Kaiserslautern, Germany

**Keywords:** amino acids, asymmetric catalysis, multicomponent reaction, palladium catalysis, Petasis reaction, sulfonamides

## Abstract

A palladium-catalyzed enantioselective three-component reaction of glyoxylic acid, sulfonamides and aryltrifluoroborates is described. This process provides modular access to the important α-arylglycine motif in moderate to good yields and enantioselectivies. The formed α-arylglycine products constitute useful building blocks for the synthesis of peptides or arylglycine-containing natural products.

## Introduction

**Figure 1:**
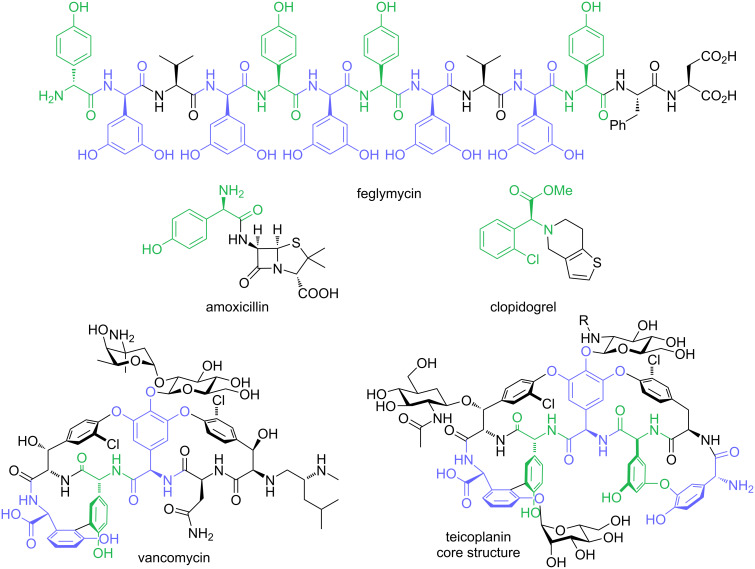
Biologically active molecules containing α-arylglycine motifs (highlighted in green and blue).

α-Amino acids play a crucial role in every aspect of our human life [1]. They are important synthetic intermediates in the chemical industry and used for the production of drugs, fertilizers, (biodegradable) polymers or nutritional supplements [2]. More importantly, α-amino acids form the backbone of all proteins and enzymes are therefore essential for almost all biological processes. In the last twenty years non-proteinogenic and chemically synthesized unnatural amino acids received increasing attention due to advances in protein-engineering and the development of protein-based therapeutics [3–4]. Among the different types of non-proteinogenic and unnatural amino acids, α-arylglycines play a particular important role. The arylglycine scaffold can be found in several well-known natural products with interesting biological properties, such as the glycopeptide antibiotics vancomycin and teicoplanin [5] or feglymycin [6], a 13mer peptide which contains nine α-arylglycines in its backbone. α-Arylglycine derivatives are used in the production of important drugs, e.g., the antiplatelet drug clopidogrel [7] or the β-lactam antibiotic amoxicillin [8] (Figure 1).

**Scheme 1:**
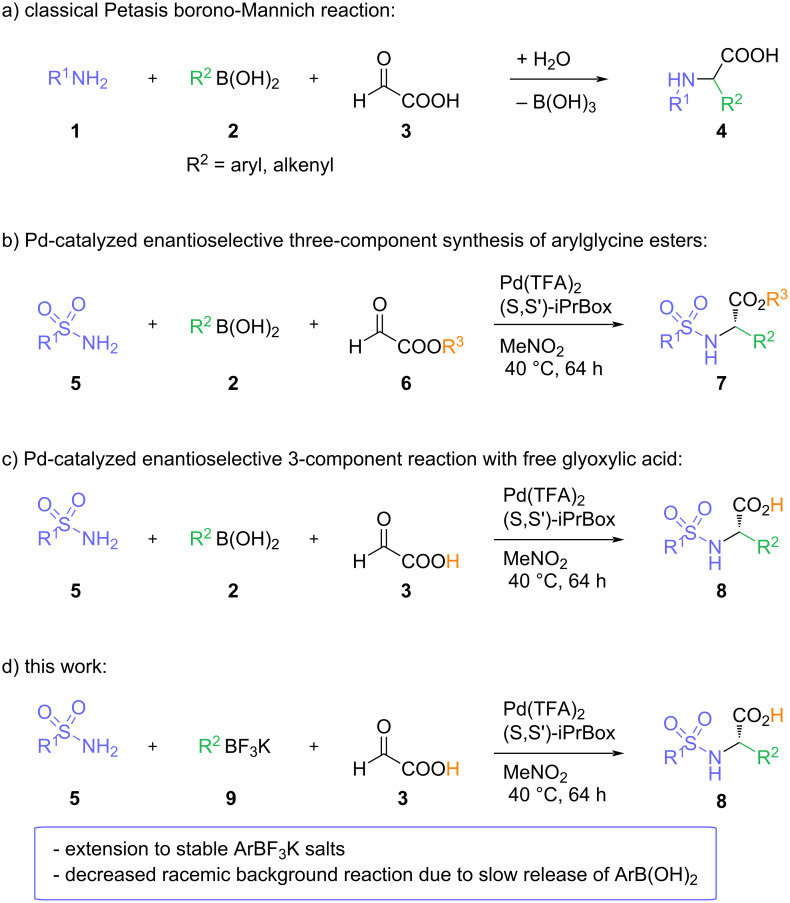
The Petasis reaction – fundamental reactivities and recent developments.

Therefore, the chemical synthesis of α-aryglycines has received considerable attention. Among the different methods introduced over time, multicomponent reactions utilizing an in situ generated reactive imine species provide a very flexible approach to the arylglycine scaffold [2,9]. The Petasis borono-Mannich reaction constitutes a prominent example for such an imine-based multicomponent reaction (Scheme 1a). The reaction of glyoxylic acid, an amine component and an arylboronic acid offers a highly modular access to arylglycines from three readily available building blocks [10–12]. The Petasis borono-Mannich reaction usually proceeds in the absence of any external catalyst via zwitterionic intermediates and an intramolecular transfer of the aryl residue form the activated boronate to the electrophilic iminium carbon, leading to the amine product as racemic mixture. Consequently, examples for asymmetric Petasis borono-Mannich reactions are rare [13] and usually rely on the utilization of chiral amine components in stoichiometric amounts [10–11].

As part of our research program utilizing the in situ generation of reactive imine species, we have disclosed iron- and bismuth-catalyzed three-component reactions for the synthesis of α-arylglycines [14–16], in which the arylboronic acid could be replaced with an electron-rich (hetero)arene as nucleophile. In parallel, we have developed palladium-catalyzed three-component reactions between arylboronic or carboxylic acids, amides or sulfonamides and different aldehyde components as attractive and broadly applicable alternative to the classical Petasis borono-Mannich reaction (Scheme 1b) [17–21]. Recently, we were able to extend these transformations to a palladium-catalyzed enantioselective synthesis of α-arylglycine bearing a free carboxylic acid functionality directly from the parent glyoxylic acids (Scheme 1c) [22]. We could show that the desired arylglycine can be synthesized in good to excellent enantioselectivities. However, depending on the nature/substitution pattern of the arylboronic acid, some of the arylglycine products could only be obtained in very low enantioselectivities. This can be attributed to a fast, uncatalyzed racemic background reaction of the boronic acids, in particular for electron-rich or sterically hindered arylboronic acids.

Herein, we report an improved version of this palladium-catalyzed enantioselective three-component reactions using aryltrifluoroborates as replacement of the arylboronic acid building block (Scheme 1d). The broader scope of this 2nd generation protocol is exploiting a slow release of the boronic acid from the aryltrifluoroborates and enables to enantioselectively synthesize of a broader variety of arylglycines, including a common building block for several biologically active compounds.

## Results and Discussion

**Scheme 2:**
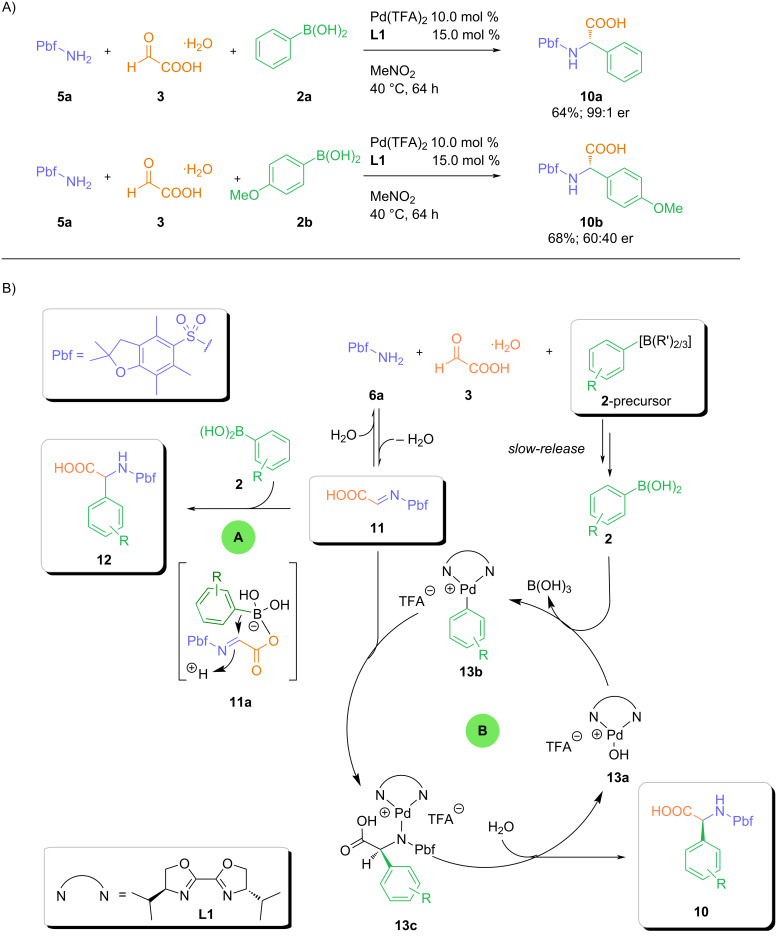
Observations from previous studies and mechanistic rationale.

During our previous studies, we observed that the enantioselectivity of the three-component coupling of glyoxylic acid (employed as its solid, easy-to-handle monohydrate) with 2,2,4,6,7-pentamethyl-2,3-dihydrobenzofuran-5-sulfonylamide, and an arylboronic acid was significantly affected by the nature of the boronic acid. Whereas the reaction with phenylboronic acid afforded the Pbf-protected [23] phenylglycine derivative **10a** in high yield and enantioselectivity, an almost racemic mixture of **10b** was obtained from the corresponding (*p*-methoxyphenyl)boronic acid (**2b**, Scheme 2a). This decrease in enantioselectivity can be attributed to a faster racemic background reaction (pathway A) via ate complex **11a** [10] of the electron-rich, more nucleophilic (*p*-methoxyphenyl)boronic acid (**2b**), which outcompetes the palladium-catalyzed pathway B (Scheme 2b). In turn, suppression or at least a significant deceleration of the uncatalyzed background reaction should lead to an increase in enantioselectivity. Decreasing the arylboronic acid to active catalyst ratio could be one possible opportunity to decrease the rate of the background reaction. Thus, we envisioned that this could be achieved by the slow generation of small amounts of the boronic acid from a suitable precursor. Among different boronic acid derivatives, we identified aryltrifluoroborates as most promising candidates for the slow generation of the corresponding arylboronic acids under our slightly acid reaction conditions [24].

**Scheme 3:**
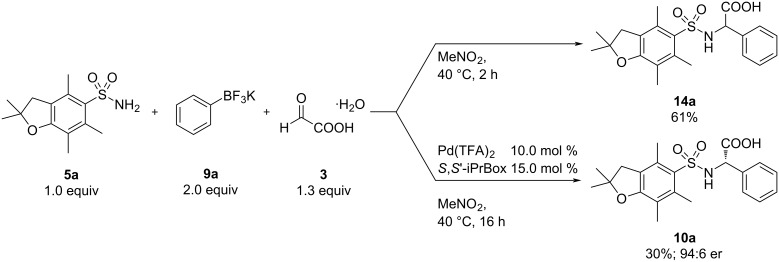
Initial experiments.

Therefore, we performed two initial control experiments. The reaction of potassium phenyltrifluoroborate with 2,2,4,6,7-pentamethyl-2,3-dihydrobenzofuran-5-sulfonylamide and glyoxylic acid in nitromethane at 40 °C in the presence and absence of our previously established Pd(TFA)_2_-*S*,*S*-iPrBox catalyst system (Scheme 3). To our delight, the palladium-catalyzed transformation afforded the desired α-arlyglycine in 30% yield and an enantiomeric ratio of 94:6. In the absence of a catalyst, the racemic product was formed in 61% yield. The comparison with the uncatalyzed reaction using free phenylboronic acid showed that the reaction of the phenyltrifluoroborate is considerably slower (61% yield after 16 h vs 89% after 2 h with PhB(OH)_2_). These preliminary studies confirmed our initial hypothesis that aryltrifluoroborates can be utilized as precursors for a slow release of the free boronic acid in our palladium-catalyzed three-component reaction.

Therefore, we started to optimize the reaction conditions for the use of potassium aryltrifluoroborate salts (Table 1). A quick survey of different solvents showed that the reaction proceeds efficiently only in nitromethane (Table 1, entry 1). Reactions in other common solvents, such as ethyl acetate, acetonitrile, tetrahydrofuran or dichloromethane led to the formation of the arylgylcine in trace amounts (Table 1, entries 2–5). Contrary to our previous report with arylboronic acids, the presence of air is highly detrimental to the reaction outcome (Table 1, entry 6). Therefore, inert conditions were employed throughout all subsequent studies. Increasing the reaction temperature to 60 °C and 80 °C furnished the desired product in increased yields of 54 and 55%, respectively, together with a slight erosion of enantioselectivity (Table 1, entries 7 and 8). Prolonging the reaction time to 64 h increased the yield to 45% without affecting the enantioselectivity (Table 1, entry 9). Also, increasing the amount of glyoxylic acid monohydrate to 2.6 equivalents furnished the arylglycine product in an improved yield of 64% and comparable enantioselectivity (Table 1, entry 10). During our experiments, we often observed partial clouding of the used glass vessel, most likely due to slow release of hydrofluoric acid, an effect which has been observed before with trifluoroborate salts [25]. Since the release of hydrofluoric acid could lead to complications with acid-labile substrates (e.g., the Pbf-protected compound **10a**) and safety issues, we decided to investigate the influence of various fluoride scavengers as additives in the three-component process [25–26]. An extensive study (not shown), revealed that most common scavengers either led to a decreased yield, a decreased stereoselectivity or a combination of both. Yet a combination of CaCO_3_, tartaric acid, and 4 Å molecular sieves, each already employed a HF scavenger by itself, did afford the desired arylglycine in high yields and enantioselectivities (Table 1, entry 11). Although, the use of this scavenger combination did lead to a slightly decreased enantioselectivity (96:4 vs 97:3), we decided to rely on these conditions in order to avoid potential troubles arising from HF release.

**Table 1:** Reaction optimization.

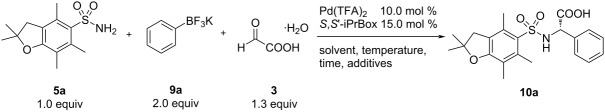			

Entry	Conditions	Yield (%)^a^	er

1	10 mol % Pd(TFA)_2_, 15 mol % ligand **L1**, 40 °C, 16 h, MeNO_2_	30	94:6
2	EtOAc instead of MeNO_2_	traces	–
3	MeCN instead of MeNO_2_	traces	–
4	THF instead of MeNO_2_	traces	–
5	CH_2_Cl_2_ instead of MeNO_2_	traces	–
6	under N_2_ atmosphere	40	98:2
7	reaction at 60 °C	54	96:4
8	reaction at 80 °C	55	94:6
9	64 h reaction time	45	98:2
10	with 2.6 equiv glyoxylic acid monohydrate	65	97:3
11	with 2.6 equiv glyoxylic acid monohydrate, 1.0 equiv CaCO_3_, 2.0 equiv tartaric acid; MS 4 Å	**79**	**96:4**

^a^Isolated yield of analytically pure product.

**Scheme 4:**
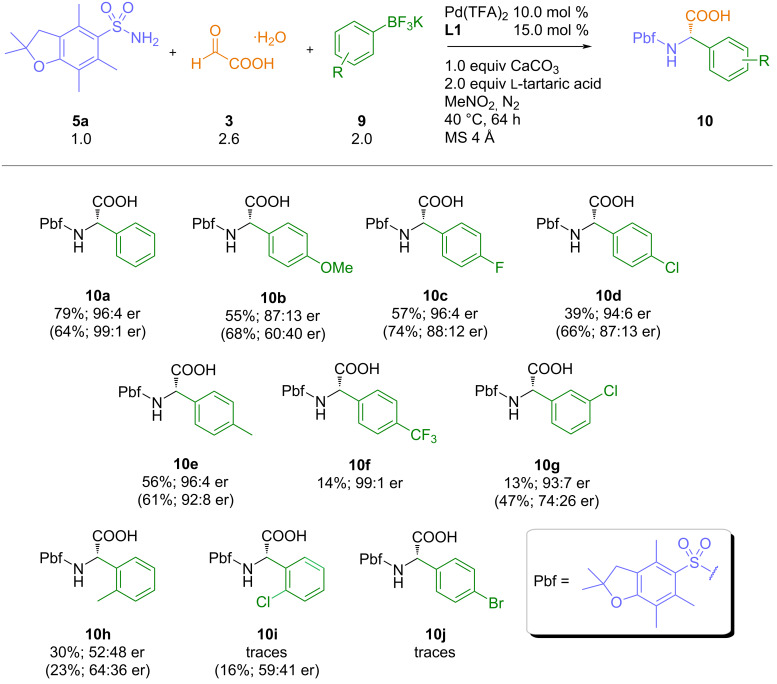
Reaction scope – aryltrifluoroborates (yields and enantiomeric ratios in parentheses refer to our previous study with the corresponding boronic acids [22]).

With the optimized conditions identified, next we studied the reaction of glyoxylic acid monohydrate and 2,2,4,6,7-pentamethyl-2,3-dihydrobenzofuran-5-sulfonylamide with different aryltrifluoroborate salts. To our delight, these three-component reactions afforded the desired arylglycines in consistently high levels of enantioselectivity, even for electron-rich aryltrifluoroborates (Scheme 4). This can be highlighted by the synthesis of the methoxy-substituted arylglycine **10b**, which was obtained in 55% yield and an enantiomeric ratio of 87:13 (compared to 68% and an enantiomeric ratio of 60:40 with the boronic acid). As in the case of arylboronic acids, reactions with a sterically hindered *ortho*-substituted trifluoroborate furnished the arylglycine product in almost racemic form. Unfortunately, reactions with aryltrifluoroborates did not proceed as efficiently as with the free boronic acid and the arylglycine products **10c**–**j** were obtained in decreased yields compared to our previous reactions with ArB(OH)_2_. We assume that a faster protodeborylation, presumably associated with the release of HF, leads to this general decrease of the isolated yields.

As already demonstrated in our previous work, the Pbf-protected arylglycine products can be directly used as building blocks for peptide synthesis [22].

**Scheme 5:**
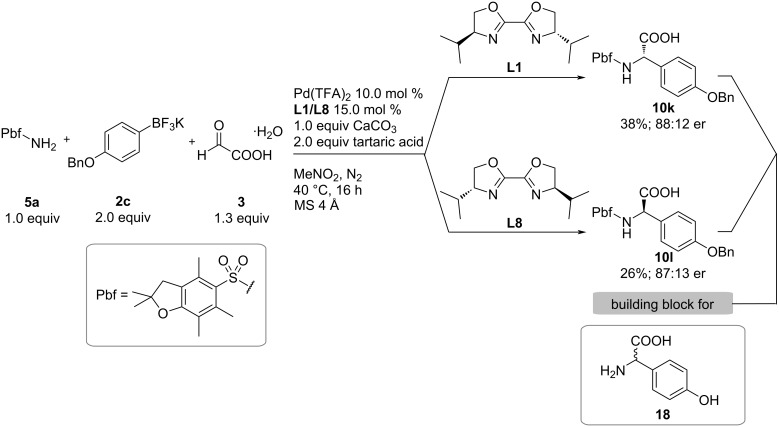
Synthesis of both enantiomers of arylglycine building block **18**.

Finally, we utilized our method for the preparation of a protected version of *p*-hydroxyphenylglycine (Scheme 5), a common structural motif in vancomycin, teicoplanin, feglymycin, and amoxicillin. Therefore, the OBn-protected aryltrifluoroborate was subjected to our standard reaction conditions, affording the desired *N*,*O*-protected (*S*)-arylglycine derivative **10k** in 38% yield and an enantiomeric ratio of 88:12. By employing the corresponding *R*,*R*-iPrBox-ligand the second enantiomer, (*R*)-arylglycine **10l** could be prepared with a similar yield and enantioselectivity.

## Conclusion

In summary, we have reported a palladium-catalyzed enantioselective three-component reaction of aryltrifluoroborates, sulfonamides, and glyoxylic acid. This method is an improved extension of our pervious protocol with arylboronic acids and provides access to enantioenriched α-arylglycines with an improved substrate diversity. It can be used for the direct synthesis of peptide-like building blocks, which can find direct application in the total synthesis of arylglycine-containing natural products. Currently, we are performing a detailed mechanistic study in order to overcome still existing limitations of the method and to provide a truly general approach to arylglycines with uniformly high yields and enantioselectivities.

## Supporting Information

File 1 Experimental section and characterization data.
