# Supplementary material for: Palladium-catalyzed enantioselective three-component synthesis of α-arylglycine derivatives from glyoxylic acid, sulfonamides and aryltrifluoroborates
Source: Beilstein J Org Chem. 2023 May 25;19:719–26. doi: 10.3762/bjoc.19.52 (PMC10241097; doi:10.3762/bjoc.19.52)
Supplement: File 1 — Experimental section and characterization data. [file Beilstein_J_Org_Chem-19-719-s001.pdf]

BEILSTEIN JOURNAL OF ORGANIC CHEMISTRY

Supporting Information

for

Palladium-catalyzed enantioselective three-component synthesis of α-arylglycine derivatives from glyoxylic acid, sulfonamides and aryltrifluoroborates

Bastian Jakob, Nico Schneider, Luca Gengenbach and Georg Manolikakes

Beilstein J. Org. Chem. **2023,** *19,* 719–726. doi:10.3762/bjoc.19.52

Experimental section and characterization data

License and Terms: This is a supporting information file under the terms of the Creative Commons Attribution License (https://creativecommons.org/licenses/by/4.0). Please note that the reuse, redistribution and reproduction in particular requires that the author(s) and source are credited and that individual graphics may be subject to special legal provisions.
The license is subject to the *Beilstein Journal of Organic Chemistry* terms and conditions: (https://www.beilstein-journals.org/bjoc/terms)

Table of contents

1 General information S2
1.1 Experimental S2
1.2 Materials S2
1.3 Analytical data and instrumentation S2
2 Preparation and analytical data S4
2.1 General procedures (GP) S4
2.2 Synthesis α-arylglycines S6
3 HPLC data S16
4 NMR data S24
5 References S34

1 General information

1.1 Experimental

Thin layer chromatography (TLC) was performed on precoated aluminum sheets (TLC silica gel 60 F_{254}). The spots were visualized by ultraviolet light, iodine or cerium(IV) ammonium molybdate. Flash column chromatography was performed using a puriflash XS 420+ flash purifier machine from Interchim with prepacked flash columns (Puriflash_Silica HP_15 µm_F0040, Puriflash PF C18HP 30 µm F0012) and the respective solvent mixture. All yields refer to the isolated yields of compounds estimated to be > 95% pure as determined by ^{1}H NMR spectroscopy.

1.2 Materials

Unless noted, all starting materials were purchased from different commercial sources and used without further purification. Sulfonamide **10** and ligand **L1** were synthesized according to known literature procedures.[1,2] Racemic products for chiral HPLC analysis were prepared according to the same typical procedures reported for the enantioselective three-component reactions by utilizing the corresponding sulfonamide (0.5 mmol), glyoxylic acid (0.65 mmol) and arylboronic acids (1.0 mmol) in nitromethane (2.0 mL) at 60 °C for 24 h.

1.3 Analytical data and instrumentation

NMR spectroscopy - Proton nuclear magnetic resonance spectra (^{1}H NMR) and carbon spectra (^{13}C NMR) were recorded at a frequency of 400 MHz (^{1}H) and 101 MHz (^{13}C), respectively. Chemical shifts are expressed as parts of million downfield shift on the δ-scale and are referenced to the solvent peak (chloroform-d_1: δ = 7.26 ppm for ^{1}H, δ = 77.16 ppm for ^{13}C; DMSO-d_6: δ = 2.50 ppm for ^{1}H, δ = 39.52 ppm for ^{13}C). ^{19}F NMR spectra were recorded proton decoupled at a frequency of 282 MHz. Chemical shifts are quoted in parts per million and are not referenced. Coupling constants (*J*) are quoted in Hz and the observed signal multiplicities are reported as follows: s = singlet, d = doublet, t = triplet, q = quartet, m = multiplet.

Mass spectrometry - Mass spectra (MS) were measured using ESI (electrospray ionization) techniques. High resolution mass spectra (HRMS) were acquired on a Waters GCT Premium using electron ionization mass spectroscopy (EI-MS-TOF).

Infrared spectroscopy - Infrared spectra (IR) were recorded on an FTIR (Fourier transform infrared spectroscopy) spectrometer including a diamond universal ATR sampling technique (attenuated total reflectance) from 4000–400 cm^{-1}. The absorption bands were reported in wave numbers (cm^{-1}).

Optical rotations - Rotation values (α) were measured with an analog-type *243B polarimeter* from *Perkin Elmer*, equipped with a sodium lamp source (589 nm), at 20 °C in a 10 cm cell and the indicated solvent. The specific rotation values are reported as $[\alpha]_\lambda^T$ (mass concentration (*c*) in g·100 mL^{-1}, solvent) and are quoted in deg·mL·dm^{-1}·g^{-1}.

Analytical chiral HPLC – Enantiomeric ratios (er) and accordingly enantiomeric excesses (ee) were determined by normal phase high performance liquid chromatographic (HPLC) analysis with a *Hewlett Packard*™ system (G1322A degasser, G1311 quadruple pump, G1316A diode array detector with visualization at 254 nm) and the use of a Chiralpak® IA, Chiralcel® OD-H or OJ-H as chiral column (4.6 mm × 25 cm) obtained from *Daicel Chemical Industries, Ltd*. Elution conditions are reported at specific compounds.

Melting points - Melting points are uncorrected.

2 Preparation and analytical data

2.1 General procedures (GP)

GP1 (initial experiments) – In a manner similar to [4] a 10 mL screw cap glass vial was charged with a magnetic stirring bar, sulfonamide **10** (134.7 mg, 0.50 mmol, 1.0 equiv), glyoxylic acid monohydrate (59.8 mg, 0.65 mmol, 1.3 equiv), potassium (phenyl)trifluoroborate (184.0 mg, 1.00 mmol, 2.0 equiv), $Pd(TFA)_2$ (16.6 mg, 50 µmol, 0.10 equiv), *S,S'*-iPrBox (**L1**, 16.8 mg, 75.0 µmol, 0.15 equiv) and nitromethane (0.25 M referring to sulfonamide, 2 mL) as solvent. Then, the vial was closed with a teflon lined screw cap and the resulting reaction mixture was stirred at 40 °C for 16 h. After cooling to room temperature, the reaction mixture was diluted with acetone and filtered through a short plug of celite and silica gel. The filter pad was rinsed with additional acetone and the combined filtrates were concentrated under reduced pressure. Purification of the crude residue by flash column chromatography afforded the analytically pure product.

GP2 (parameter optimization) – In a manner similar to [4] a 8 mL glass vial with a ground glass joint was charged with a magnetic stirring bar, sulfonamide **10** (134.7 mg, 0.50 mmol, 1.0 equiv), glyoxylic acid monohydrate (59.8 mg, 0.65 mmol, 1.3 equiv), potassium phenyltrifluoroborate (184.0 mg, 1.00 mmol, 2.0 equiv), $Pd(TFA)_2$ (16.6 mg, 50 µmol, 0.10 equiv), *S,S'*-iPrBox L1 (16.8 mg, 75.0 µmol, 0.15 equiv). The glass vial was closed with a rubber septum, evacuated, and backfilled with nitrogen twice before adding nitromethane (0.25 M referring to sulfonamide, 2 mL) as solvent. The resulting reaction mixture was stirred at 40 °C for 16 h. After cooling to room temperature, the reaction mixture was diluted with acetone and filtered through a short plug of celite and silica gel. The filter pad was rinsed with additional acetone and the combined filtrates were concentrated under reduced pressure. Purification of the crude residue by flash column chromatography afforded the analytically pure product.

GP3 (BF_3K salt variation) – An 8 mL glass vial with a ground glass joint was charged with a magnetic stirring bar, sulfonamide **10** (134.7 mg, 0.50 mmol, 1.0 equiv), glyoxylic acid monohydrate (119.6 mg, 1.30 mmol, 2.6 equiv), potassium aryltrifluoroborate (1.00 mmol, 2.0 equiv), $Pd(TFA)_2$ (16.6 mg, 50 µmol, 0.10 equiv), *S,S'*-iPrBox L1 (16.8 mg, 75.0 µmol, 0.15 equiv), $CaCO_3$ (50.1 mg, 0.5 mmol, 1.0 equiv), tartaric acid (150.9 mg, 1.0 mmol, 2.0 equiv) and molecular sieves 4 Å (200 mg). The glass vial was closed with a rubber septum, evacuated and backfilled with nitrogen twice before adding nitromethane (0.25 M referring to sulfonamide, 2 mL) as solvent. The resulting reaction mixture was stirred at 40 °C for 16 h. After cooling to room temperature, the reaction mixture was diluted with

acetone and filtered through a short plug of celite and silica gel. The filter pad was rinsed with additional acetone and the combined filtrates were concentrated under reduced pressure. Purification of the crude residue by flash column chromatography afforded the analytically pure product.

GP4 (BF_3K salt synthesis) – A 100 mL round-bottomed flask was charged with a magnetic stirring bar, boronic acid (8.2 mmol, 1.0 equiv) and 40 mL MeCN. Afterwards an aqueous KF solution (3.3 mL, 10 M, 32.8 mmol, 4.0 equiv) was added and the mixture stirred at room temperature for 15 minutes. Then, tartaric acid solution (33.5 mL, 1 M in THF, 33.6 mmol, 2.05 equiv) was slowly dropped into the reaction mixture and stirred for additional 30 minutes. The reaction mixture was filtered and washed three times with 15 mL MeCN each. The solution was concentrated to 20 mL in vacuo and Et_2O was added until the product precipitated. The product was filtered again, washed with Et_2O and dried in an oil pump vacuum.

2.2 Synthesis α-arylglycines

(*S*)-2-((2,2,4,6,7-Pentamethyl-2,3-dihydrobenzofuran)-5-sulfonamido)-2-phenylacetic acid (**10a**)

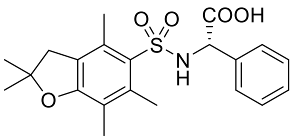

10a

Prepared according to **GP3** from potassium phenyltrifluoroborate (184.0 mg, 1.00 mmol, 2.0 equiv). Purification by reversed phase column chromatography (H_2O/MeCN + 0.1 vol % TFA = 9:1 → 2:8) and freeze drying afterwards afforded product **10a** as a colorless solid (159 mg, 79%). Analytical data match those reported in the literature. [4]

$[\alpha]_D^{20}$ = +91.8 (c 0.1, $CHCl_3$)

e.r = 96:4 [HPLC conditions: Chiralcel ® IA column, *n*-hexane/ethanol/TFA = 9:1:0.1, flow rate = 0.7 mL/min, t_R (minor) = 21.3 min and t_R (major) = 23.1 min].

R_f (*n*-hexane/acetone/AcOH = 2:1:0.1) 0.31

^{1}H NMR (400 MHz, $CDCl_3$) δ = 7.26 – 7.13 (m, 5H), 5.63 (d, *J* = 8 Hz, 1H), 4.99 (d, *J* = 4 Hz, 1H), 2.88 (t, *J* = 16 Hz, 2H), 2.46 (s, 3H), 2.38 (s, 3H), 2.02 (s, 3H), 1.45 (s, 6H) ppm.

^{13}C NMR (101 MHz, $CDCl_3$) δ = 174.1, 159.85, 139.50, 134.79, 134.12, 128.65, 128.56, 127.73, 127.15, 125.03, 117.99, 86.85, 58.93, 43.04, 28.55, 28.53, 19.28, 17.66, 12.38 ppm.

MS (APCI) m/z calcd for $C_{21}H_{25}NO_5S$ 404.5 $[M+H]^+$, found 404.2 $[M+H]^+$

HRMS (TOF MS EI+) m/z calcd for $C_{21}H_{25}NO_5S$ 403.1453 $[M^+]$, found 403.1472 $[M^+]$

(*S*)-2-(4-Methoxyphenyl)-2-((2,2,4,6,7-pentamethyl-2,3-dihydrobenzofuran)-5-sulfonamido)acetic acid (**10b**)

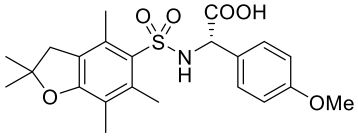

10b

Prepared according to **GP3** from potassium 4-methoxyphenyltrifluorborate (214.0 mg, 1.00 mmol, 2.0 equiv). Purification by reversed phase column chromatography (H_2O/MeCN + 0.1 vol % TFA = 9:1 → 2:8) and freeze drying afterwards afforded product **10b** as a colorless solid (121 mg, 55%). Analytical data match those reported in the literature. [4]

$[\alpha]_D^{20}$ = +74.5 (c 0.1, $CHCl_3$)

e.r = 88:12 [HPLC conditions: Chiralcel ® IA column, *n*-hexane/ethanol/TFA = 9:1:0.1, flow rate = 0.7 mL/min, t_R (minor) = 25.2 min and t_R (major) = 29.4 min].

R_f (*n*-hexane/acetone/AcOH = 2:1:0.1) 0.31

^{1}H NMR (400 MHz, $CDCl_3$) δ = 7.05 (d, *J* = 8 Hz, 2H), 6.71 (d, *J* = 8 Hz, 2H), 5.60 (d, *J* = 4 Hz, 1H), 4.93 (d, 1H, *J* = 8 Hz), 3.74 (s, 3H), 2.88 (s, 2H), 2.45 (s, 3H), 2.38 (s, 3H), 2.01 (s, 3H), 1.45 (s, 6H) ppm.

^{13}C NMR (101 MHz, $CDCl_3$) δ =174.50, 159.79, 159.77, 139.42, 134.07, 128.39, 127.91, 126.81, 125.02, 117.95, 113.88, 86.84, 58.44, 55.24, 43.06, 28.50, 28.44, 19.29, 17.67, 12.37 ppm.

MS (APCI) m/z calcd for $C_{22}H_{27}NO_6S$ 434.2 $[M+H]^+$, found 434.3 $[M+H]^+$

HRMS (TOF MS EI+) m/z calcd for $C_{22}H_{27}NO_6S$ 433.1559 $[M^+]$, found 433.1567 $[M^+]$

(*S*)-2-(4-Fluorophenyl)-2-((2,2,4,6,7-pentamethyl-2,3-dihydrobenzofuran)-5-sulfonamido)acetic acid (**10c**)

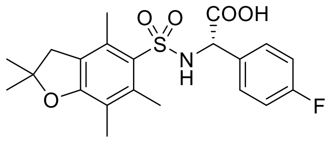

10c

Prepared according to **GP3** from (4-fluorophenyl)boronic acid (139.9 mg, 1.00 mmol, 2.0 equiv). Purification by reversed phase column chromatography (H_2O/MeCN + 0.1 vol % TFA = 9:1 → 2:8) and freeze drying afterwards afforded product **10c** as a colorless solid (155 mg, 74%). Analytical data match those reported in the literature. [4]

$[\alpha]_D^{20}$ = +78.3 (c 0.1, $CHCl_3$)

e.r = 88:12 [HPLC conditions: Chiralcel ® IA column, *n*-hexane/ethanol/TFA = 9:1:0.1, flow rate = 0.7 mL/min, t_R (minor) = 18.9 min and t_R (major) = 22.6 min].

R_f (*n*-hexane/acetone/AcOH = 2:1:0.1) 0.34

^{1}H NMR (400 MHz, $CDCl_3$) δ = 7.13 (dd, *J* = 8 Hz, 2H), 6.88 (t, *J* = 8 Hz, 2H) 5.68 (d, *J* = 8 Hz, 1H), 5.00 (d, 1H, *J* = 4 Hz), 2.88 (s, 2H), 2.47 (s, 3H), 2.35 (s, 3H), 2.03 (s, 3H), 1.46 (d, *J* = 4 Hz, 6H) ppm.

^{13}C NMR (101 MHz, $CDCl_3$) δ = 173.79, 162.72 (d, *J* = 247.5 Hz) 159.92, 139.42, 134.08, 130.68 (d, *J* = 3.0 Hz), 129.04 (d, *J* = 8.1 Hz), 127.71, 125.07, 118.06, 115.45 (d, *J* = 21.2 Hz), 86.93, 58.27, 43.03, 28.52, 28.46, 19.26, 17.67, 12.39 ppm.

^{19}F NMR (376 MHz, $CDCl_3$) δ = -112.59 ppm.

MS (APCI) m/z calcd for $C_{21}H_{24}FNO_5S$ 422.2 $[M+H]^+$, found 422.3 $[M+H]^+$

HRMS (TOF MS EI+) m/z calcd for $C_{21}H_{24}FNO_5S$ 421.1359 $[M^+]$, found 421.1358 $[M^+]$

(*S*)-2-(4-Chlorophenyl)-2-((2,2,4,6,7-pentamethyl-2,3-dihydrobenzofuran)-5-sulfonamido)acetic acid (**10d**)

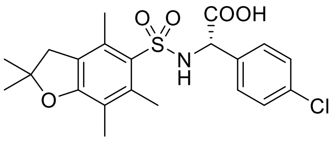

10d

Prepared according to **GP3** from (4-chlorophenyl)boronic acid (156.4 mg, 1.00 mmol, 2.0 equiv). Purification by reversed phase column chromatography (H_2O/MeCN + 0.1 vol % TFA = 9:1 → 2:8) and freeze drying afterwards afforded product **10d** as a colorless solid (145 mg, 66%). Analytical data match those reported in the literature. [4]

$[\alpha]_D^{20}$ = +81.7 (c 0.1, $CHCl_3$)

e.r = 87:13 [HPLC conditions: Chiralcel ® IA column, *n*-hexane/ethanol/TFA = 9:1:0.1, flow rate = 0.7 mL/min, t_R (minor) = 19.4 min and t_R (major) = 23.8 min].

R_f (*n*-hexane/acetone/AcOH = 2:1:0.1) 0.36

^{1}H NMR (400 MHz, $CDCl_3$) δ = 7.16 (d, *J* = 8 Hz, 2H), 7.08 (d, *J* = 8 Hz, 2H), 5.77 (d, *J* = 8 Hz, 1H), 4.99 (d, *J* = 8 Hz, 1H), 2.88 (s, 2H), 2.45 (s, 3H), 2.34 (s, 3H), 2.03 (s, 3H), 1.46 (d, *J* = 8 Hz, 6H) ppm.

^{13}C NMR (101 MHz, $CDCl_3$) δ =173.78, 159.98, 139.43, 134.65, 134.08, 133.33, 128.60, 127.60, 125.11, 118.10, 86.98, 58.40, 43.01, 28.51, 28.47, 19.25, 17.67, 12.38 ppm.

MS (APCI) m/z calcd for $C_{21}H_{24}ClNO_5S$ 438.1 $[M+H]^+$, found 438.3 $[M+H]^+$

HRMS (TOF MS EI+) m/z calcd for $C_{21}H_{24}ClNO_5S$ [^{35}Cl] 437.1064 [M^+], found 437.1062 [M^+]; [^{37}Cl] 439.1034 [M^+], found 439.1053 [M^+]

(*S*)-2-((2,2,4,6,7-Pentamethyl-2,3-dihydrobenzofuran)-5-sulfonamido)-2-(*p*-tolyl)acetic acid (**10e**)

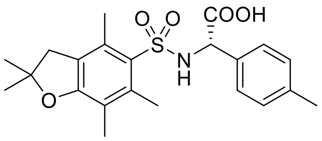

10e

Prepared according to **GP3** from potassium *p*-tolyltrifluoroborate (136.0 mg, 1.00 mmol, 2.0 equiv). Purification by reversed phase column chromatography (H_2O/MeCN + 0.1 vol % TFA = 9:1 → 2:8) and freeze drying afterwards afforded product **10e** as a colorless solid (117 mg, 56%). Analytical data match those reported in the literature. [4]

$[\alpha]_D^{20}$ = +89.8 (c 0.1, $CHCl_3$)

e.r = 96:4 [HPLC conditions: Chiralcel ® IA column, *n*-hexane/ethanol/TFA = 9:1:0.1, flow rate = 0.7 mL/min, t_R (minor) = 22.4 min and t_R (major) = 24.8 min].

R_f (*n*-hexane/acetone/AcOH = 2:1:0.1) 0.36

^{1}H NMR (400 MHz, $CDCl_3$) δ = 7.04 – 6.99 (m, 4fH), 5.59 (d, 1H), 4.93 (d, *J* = 4 Hz, 1H), 2.90 (s, 2H), 2.44 (s, 3H), 2.39 (s, 3H), 2.27 (s, 3H) 2.01 (s, 3H), 1.46 (d, *J* = 4 Hz, 6H) ppm.

^{13}C NMR (101 MHz, $CDCl_3$) δ = 174.53, 159.81, 139.50, 138.63, 134.12, 131.84, 129.23, 127.79, 127.01, 125.02, 117.96, 86.81, 58.71, 43.08, 28.49, 21.12, 19.29, 17.67, 12.35 ppm.

MS (APCI) m/z calcd for $C_{22}H_{27}NO_5S$ 418.2 $[M+H]^+$, found 418.3 $[M+H]^+$

HRMS (TOF MS EI+) m/z calcd for $C_{22}H_{27}NO_5S$ 417.1610 $[M^+]$, found 417.1605 $[M^+]$

(*S*)-2-((2,2,4,6,7-Pentamethyl-2,3-dihydrobenzofuran)-5-sulfonamido)-2-(4-(trifluoromethyl)phenyl)acetic acid (**10f**)

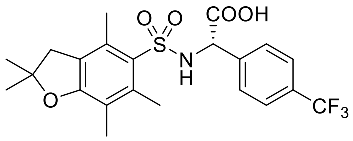

10f

Prepared according to **GP3** from potassium (*p*-trifluoromethylphenyl)trifluoroborate (252.0 mg, 1.00 mmol, 2.0 equiv). Purification by reversed phase column chromatography (H_2O/MeCN + 0.1 vol % TFA = 9:1 → 2:8) and freeze drying afterwards afforded product **10f** as a colorless solid (32 mg, 14%).

$[\alpha]_D^{20}$ = +120.4 (c 0.1, $CHCl_3$)

e.r. = 99:1 HPLC conditions: Chiralcel ® IA column, *n*-hexane/ethanol/TFA = 9:1:0.1, flow rate = 0.7 mL/min, t_R (minor) = 18.5 min and t_R (major) = 21.3 min].

R_f (*n*-hexane/acetone/AcOH = 2:1:0.1) 0.36

m.p. 161-163 °C

^{1}H NMR (400 MHz, $CDCl_3$) δ = 7.42 (d, *J* = 8 Hz, 2H), 7.28 (d, *J* = 8 Hz, 2H), 6.10 (d, *J* = 16 Hz, 1H), 5.06 (d, *J* = 8 Hz, 1H), 2.84 (s, 2H), 2.42 (s, 3H), 2.33 (s, 3H), 1.98 (s, 3H), 1.44 (d, *J* = 4 Hz, 6H) ppm.

^{13}C NMR (101 MHz, $CDCl_3$) δ = 173.30, 160.05, 139.54, 138.95, 134.13, 130.74 (q, *J* = 33.3 Hz), 127.72, 127.25, 125.28 (q, *J* = 3.9 Hz), 125.14, 123.73 (q, *J* = 272.7 Hz), 118.12, 87.00, 58.69, 42.95, 28.43, 28.39, 19.21, 17.63, 12.28 ppm.

^{19}F NMR (376 MHz, $CDCl_3$) δ = -62.67 ppm.

MS (APCI) m/z calcd for $C_{22}H_{24}F_3NO_5S$ 472.2 $[M+H]^+$, found 472.3 $[M+H]^+$

HRMS (TOF MS EI+) m/z calcd for $C_{22}H_{24}F_3NO_5S$ 471.1327 [M^+], found 471.1337 [M^+]

IR (ν in cm^{-1}): 3375, 2970, 2929, 1728, 1694, 1577, 1455, 1368, 1142, 1091, 989, 888, 850, 782, 636, 617, 562, 537.

(*S*)-2-(3-Chlorophenyl)-2-((2,2,4,6,7-pentamethyl-2,3-dihydrobenzofuran)-5-sulfonamido)acetic acid (**10g**)

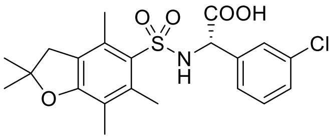

10g

Prepared according to **GP3** from potassium (3-chlorophenyl)trifluoroborate (218.5 mg, 1.00 mmol, 2.0 equiv). Purification by reversed phase column chromatography (H_2O/MeCN + 0.1 vol % TFA = 9:1 → 2:8) and freeze drying afterwards afforded product **10g** as a colorless solid (28 mg, 13%). Analytical data match those reported in the literature. [4]

$[\alpha]_D^{20}$ = +86.4 (c 0.1, $CHCl_3$)

e.r = 97:3 [HPLC conditions: Chiralcel ® IA column, *n*-hexane/ethanol/TFA = 9:1:0.1, flow rate = 0.7 mL/min, t_R (minor) = 18.8 min and t_R (major) = 21.1 min].

R_f (*n*-hexane/acetone/AcOH = 2:1:0.1) 0.42

^{1}H NMR (400 MHz, $CDCl_3$) δ = 7.18 – 7.05 (m, 4H), 5.73 (d, *J* = 8 Hz, 1H), 5.02 (d, 1H, *J* = 4 Hz), 2.87 (q, *J* = 10 Hz, 2H), 2.46 (s, 3H), 2.35 (s, 3H), 2.02 (s, 3H), 1.45 (s, 6H) ppm.

^{13}C NMR (101 MHz, $CDCl_3$) δ = 172.78, 159.92, 139.33, 136.66, 134.39, 133.97, 129.61, 128.65, 127.65, 127.36, 125.55, 125.07, 118.16, 86.88, 58.50, 43.00, 28.60, 28.57, 19.24, 17.66, 12.40 ppm.

MS (APCI) m/z calcd for $C_{21}H_{24}ClNO_5S$ 438.1 $[M+H]^+$, found 438.3 $[M+H]^+$

HRMS (TOF MS EI+) m/z calcd for $C_{21}H_{24}ClNO_5S$ [^{37}Cl] 439.1034 [M^+], found 439.1052 [M^+]

(*S*)-2-((2,2,4,6,7-Pentamethyl-2,3-dihydrobenzofuran)-5-sulfonamido)-2-(*o*-tolyl)acetic acid (**10h**)

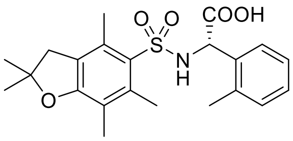

10h

Prepared according to **GP3** from potassium (2-methylphenyl)trifluoroborate (198.0 mg, 1.00 mmol, 2.0 equiv). Purification by reversed phase column chromatography (H_2O/MeCN + 0.1 vol % TFA = 9:1 → 2:8) and freeze drying afterwards afforded product **10h** as a colorless solid (63 mg, 30%). Analytical data match those reported in the literature. [4]

$[\alpha]_D^{20}$ = +0.0 (c 0.1, $CHCl_3$)

e.r = 50:50 [HPLC conditions: Chiralcel ® IA column, *n*-hexane/ethanol/TFA = 9:1:0.1, flow rate = 0.7 mL/min, t_R (minor) = 20.6 min and t_R (major) = 23.4 min].

R_f (*n*-hexane/acetone/AcOH = 2:1:0.1) 0.29

^{1}H NMR (400 MHz, $CDCl_3$) δ = 7.14– 6.98f (m, 4H), 5.60 (d, *J* = 4 Hz, 1H), 5.24 (d, *J* = 4 Hz, 1H), 2.86 (s, 2H), 2.45 (s, 3H), 2.36 (s, 3H), 2.26 (s, 3H), 2.00 (s, 3H), 1.45 (d, *J* = 4 Hz, 6H) ppm.

^{13}C NMR (101 MHz, $CDCl_3$) δ = 174.42, 159.76, 139.53, 136.37, 134.07, 133.39, 130.72, 128.54, 127.80, 126.91, 126.11, 124.99, 117.95, 86.81, 55.52, 43.02, 28.50, 19.20, 19.07, 17.65, 12.35 ppm.

MS (APCI) m/z calcd for $C_{22}H_{27}NO_5S$ 418.2 $[M+H]^+$, found 418.3 $[M+H]^+$

HRMS (TOF MS EI+) m/z calcd for $C_{22}H_{27}NO_5S$ 417.1610 $[M^+]$, found 417.1622 $[M^+]$

(*S*)-2-(4-(Benzyloxy)phenyl)-2-((2,2,4,7-tetramethyl-2,3-dihydrobenzofuran)-5-sulfonamido)acetic acid (**10k**)

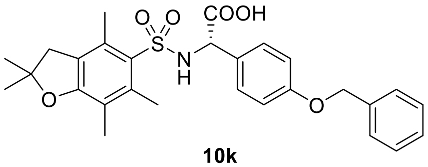

10k

Prepared according to **GP3** from potassium (4-benzyloxyphenyl)trifluoro borate (290.1 mg, 1.00 mmol, 2.0 equiv). Purification by reversed phase column chromatography (H_2O/MeCN + 0.1 vol % TFA = 9:1 → 2:8) and freeze drying afterwards afforded product **10k** as a colorless solid (96 mg, 38%).

$[\alpha]_D^{20}$ = +64.0 (c 0.1, $CHCl_3$)

e.r. = 88:12 [HPLC conditions: Chiralcel ® IA column, *n*-hexane/ethanol/TFA = 9:1:0.1, flow rate = 0.7 mL/min, t_R (minor) = 28.3 min and t_R (major) = 29.7 min].

R_f (*n*-Hexan/Aceton/AcOH = 2:1:0.1) 0.31

m.p. 158-161 °C

^{1}H NMR (400 MHz, $CDCl_3$) δ = 7.39-7.33 (m, 5H), 7.06 (d, *J* = 8 Hz, 2H), 6.79 (d, *J* = 8 Hz, 2H) 5.60 (d, *J* = 4 Hz, 1H), 4.98 (d, *J* = 8 Hz, 2H), 2.90 (s, 2H), 2.45 (s, 3H), 2.39 (s, 3H), 2.02 (s, 3H), 1.45 (s, 6H) ppm.

^{13}C NMR (101 MHz, $CDCl_3$) δ = 174.46, 159.81, 159.06, 139.48, 136.57, 134.11, 128.64, 128.44, 127.87, 127.46, 127.13, 125.06, 117.97, 114.77, 86.86, 70.04, 58.43, 43.08, 28.51, 19.30, 17.68, 12.41 ppm.

MS (APCI) m/z calcd for $C_{28}H_{31}NO_6S$ 510.6 $[M+H]^+$, found 510.4 $[M+H]^+$

HRMS (TOF MS EI+) m/z calcd for $C_{28}H_{31}NO_6S$ 509.1872 $[M^+]$, found 509.1883 $[M^+]$

IR (v in cm^{-1}): 2970, 1738, 1575, 1511, 1371, 1304, 1231, 1218, 1136, 1088, 780, 732, 638, 615, 529.

(*R*)-2-(4-(Benzyloxy)phenyl)-2-((2,2,4,7-tetramethyl-2,3-dihydrobenzofuran)-5-sulfonamido)acetic acid (**10l**)

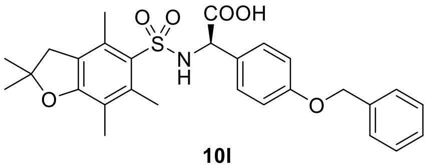

10l

Prepared according to **GP3** from potassium (4-benzyloxyphenyl)trifluoroborate (290.1 mg, 1.00 mmol, 2.0 equiv). Purification by reversed phase column chromatography (H_2O/MeCN + 0.1 vol % TFA = 9:1 → 2:8) and freeze drying afterwards afforded product **10l** as a colorless solid (67 mg, 26%).

$[\alpha]_D^{20}$ = -74.0 (c 0.1, $CHCl_3$)

e.r. = 87:13 [HPLC conditions: Chiralcel ® IA column, *n*-hexane/ethanol/TFA = 9:1:0.1, flow rate = 0.7 mL/min, t_R (minor) = 39.0 min and t_R (major) = 36.2 min].

R_f (*n*-Hexan/Aceton/AcOH = 2:1:0.1) 0.31

m.p. 159-162 °C

^{1}H NMR (400 MHz, $CDCl_3$) δ = 7.05 (d, *J* = 8 Hz, 2H), 6.71 (d, *J* = 8 Hz, 2H), 5.62 (d, *J* = 4 Hz, 1H), 4.93 (d, 1H, *J* = 8 Hz), 3.74 (s, 3H), 2.88 (s, 2H), 2.45 (s, 3H), 2.37 (s, 3H), 2.01 (s, 3H), 1.45 (s, 6H) ppm.

MS (APCI) m/z calcd for $C_{28}H_{31}NO_6S$ 510.6 $[M+H]^+$, found 510.4 $[M+H]^+$

HRMS (TOF MS EI+) m/z calcd for $C_{28}H_{31}NO_6S$ 509.1872 $[M^+]$, found 509.1887 $[M^+]$

IR (ν in cm^{-1}): 2970, 1738, 1575, 1511, 1371, 1304, 1231, 1218, 1136, 1088, 780, 732, 638, 615, 529.

3 HPLC data

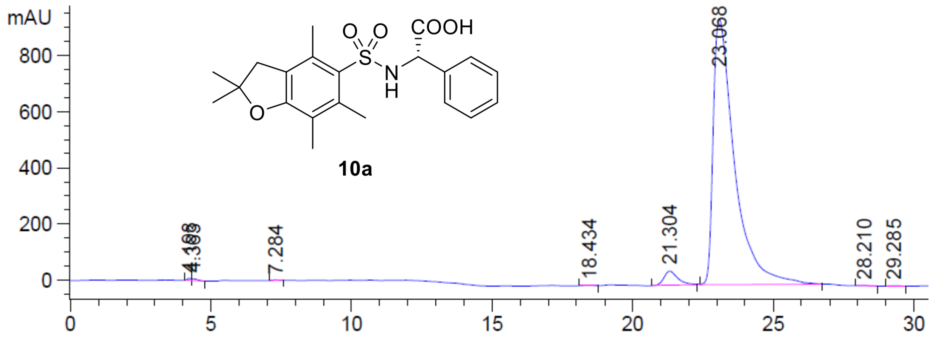

Signal 2: DAD1 B, Sig=254,16 Ref=380,100

Peak #	RetTime [min]	Type	Width [min]	Area [mAU*s]	Height [mAU]	Area %
1	4.198	BV	0.1411	72.41557	7.74436	0.1376
2	4.363	VV	0.1953	99.94223	6.65119	0.1899
3	7.284	BB	0.1635	25.78566	2.32256	0.0490
4	18.434	BV	0.2799	33.17374	1.54863	0.0630
5	21.304	BB	0.5308	1845.60498	51.40861	3.5073
6	23.068	BB	0.7884	5.02396e4	947.75934	95.4717
7	28.210	BB	0.2292	23.31352	1.27601	0.0443
8	29.285	BB	0.2751	25.42038	1.26126	0.0483
9	31.432	BB	0.2938	27.37650	1.33170	0.0520
10	32.604	BB	0.3343	37.34045	1.68655	0.0710
11	33.924	BB	0.3303	39.58357	1.64728	0.0752
12	35.147	BB	0.2903	31.96850	1.48986	0.0608
13	37.327	BB	0.2994	27.08632	1.30920	0.0515
14	38.545	BB	0.3028	35.43021	1.59707	0.0673
15	46.007	BB	0.2718	29.43589	1.33230	0.0559
16	47.168	BB	0.3023	29.01262	1.36311	0.0551

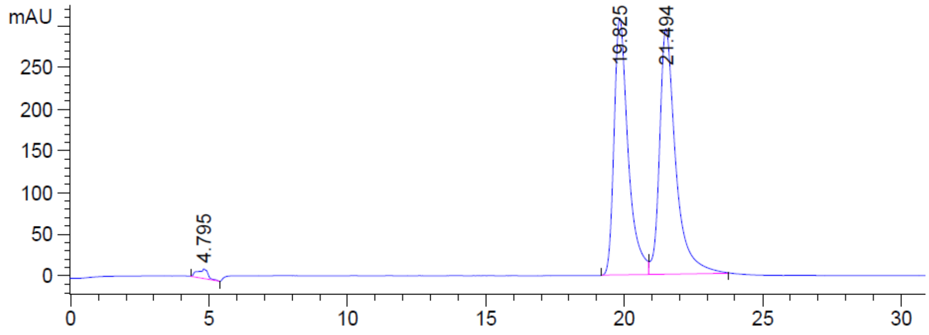

Peak #	RetTime [min]	Type	Width [min]	Area [mAU*s]	Height [mAU]	Area %
1	4.795	BV	0.3316	307.34262	11.65567	1.3259
2	19.825	BV	0.5289	1.09077e4	308.19196	47.0582
3	21.494	VB	0.6017	1.19642e4	294.68765	51.6158

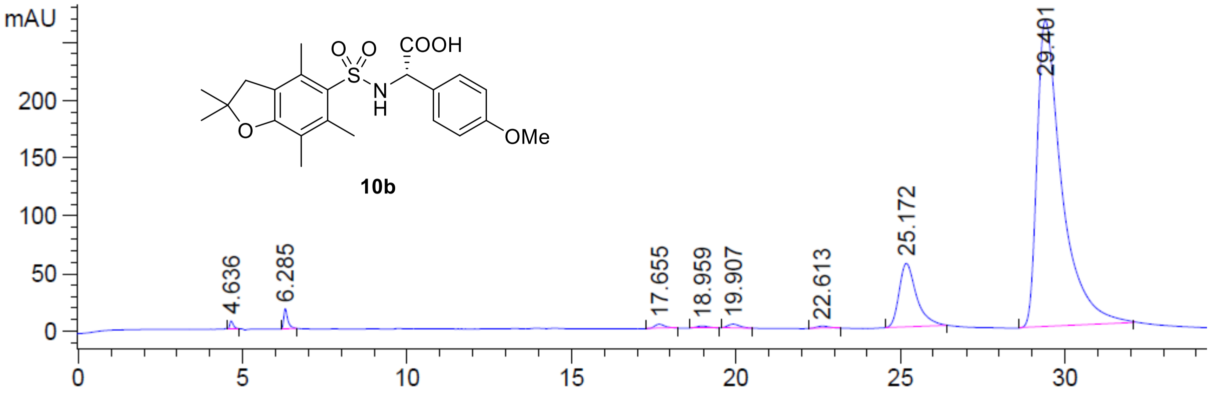

Signal 2: DAD1 B, Sig=254,16 Ref=380,100

Peak #	RetTime [min]	Type	Width [min]	Area [mAU*s]	Height [mAU]	Area %
1	4.636	VB	0.1183	53.06461	6.85620	0.3217
2	6.285	BB	0.1288	153.99100	17.48238	0.9334
3	17.655	BB	0.3266	87.18548	3.73143	0.5285
4	18.959	BB	0.3481	50.30979	1.83377	0.3050
5	19.907	BB	0.3805	96.15298	3.52167	0.5828
6	22.613	BB	0.3465	54.84767	1.93602	0.3325
7	25.172	BB	0.5533	2058.32520	55.16533	12.4765
8	29.401	BB	0.7693	1.39437e4	265.26846	84.5197

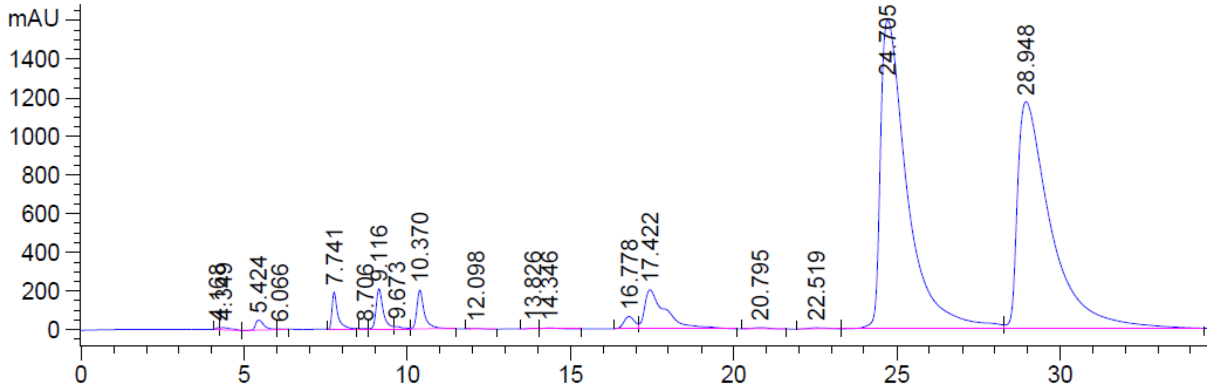

Signal 2: DAD1 B, Sig=254,16 Ref=380,100

Peak #	RetTime [min]	Type	Width [min]	Area [mAU*s]	Height [mAU]	Area %
1	4.168	BV	0.1132	82.02338	11.23207	0.0415
2	4.349	VV	0.3077	305.31033	12.92062	0.1547
3	5.424	VB	0.2885	992.92761	52.53289	0.5030
4	6.066	BB	0.1972	55.68750	3.75167	0.0282
5	7.741	BB	0.1814	2395.23901	192.18964	1.2133
6	8.706	BV	0.2108	58.75045	3.92189	0.0298
7	9.116	VV	0.2266	3200.89844	209.14458	1.6214
8	9.673	VV	0.2479	232.84340	13.06846	0.1179
9	10.370	VB	0.2382	3289.87109	201.78976	1.6665
10	12.098	BB	0.2589	46.99249	2.60005	0.0238
11	13.826	BV	0.3355	92.35009	3.80041	0.0468
12	14.346	VB	0.4744	196.36867	5.75023	0.0995
13	16.778	BV	0.3580	1479.89856	63.95156	0.7497
14	17.422	VB	0.6206	9127.63574	202.22566	4.6237
15	20.795	BB	0.5354	270.13916	7.47817	0.1368
16	22.519	BV	0.5264	277.62607	6.32148	0.1406
17	24.705	VV	0.8434	9.15133e4	1599.83997	46.3570
18	28.948	VB	1.0315	8.37920e4	1175.74207	42.4457

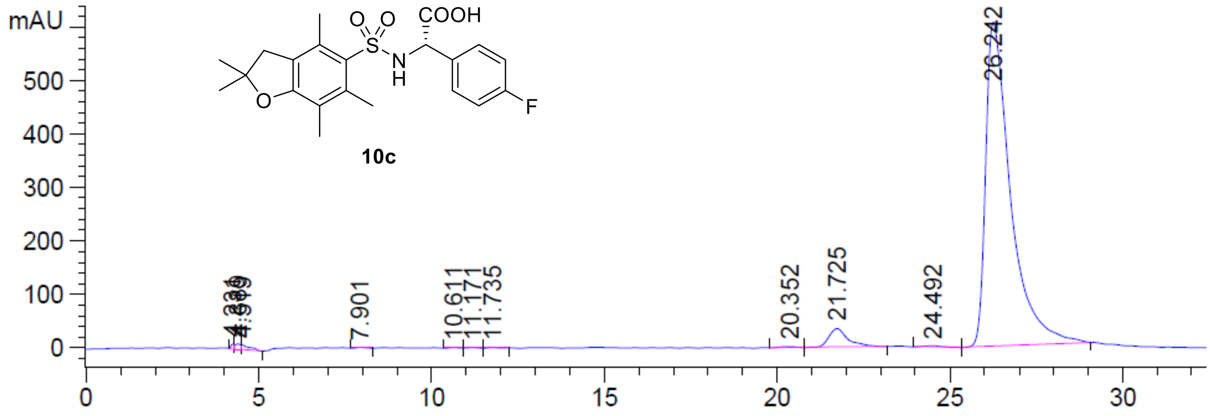

Signal 2: DAD1 B, Sig=254,16 Ref=380,100

Peak #	RetTime [min]	Type	Width [min]	Area [mAU*s]	Height [mAU]	Area %
1	4.231	BV	0.1172	79.68301	9.97425	0.2451
2	4.389	VV	0.1425	111.25517	11.14017	0.3422
3	4.519	VV	0.2307	172.14340	9.44154	0.5294
4	7.901	BV	0.2327	25.82013	1.61337	0.0794
5	10.611	BV	0.2170	19.46048	1.16426	0.0599
6	11.171	VV	0.2349	19.71332	1.04123	0.0606
7	11.735	VB	0.2481	20.39672	1.02523	0.0627
8	20.352	BV	0.4181	74.25783	2.17641	0.2284
9	21.725	VB	0.5779	1354.17615	34.80343	4.1648
10	24.492	BV	0.4176	98.18373	2.78273	0.3020
11	26.242	VB	0.7664	3.05395e4	607.59558	93.9255

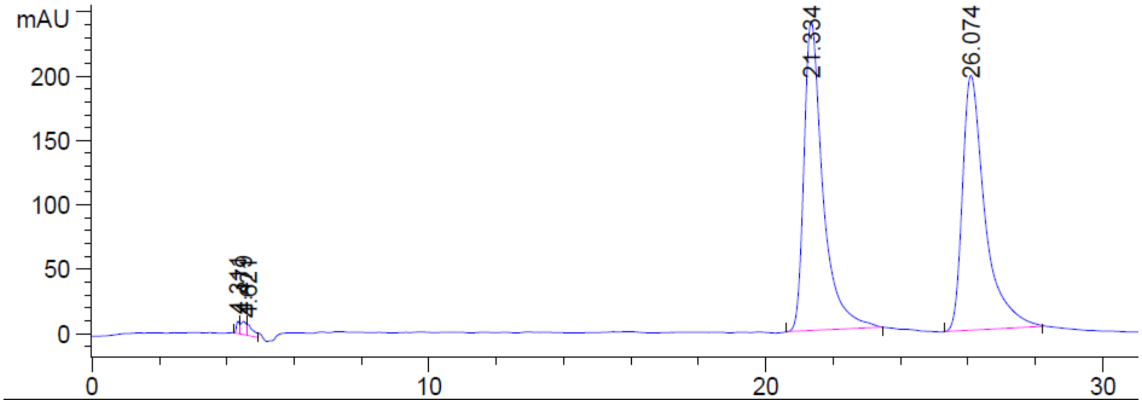

Signal 2: DAD1 B, Sig=254,16 Ref=380,100

Peak #	RetTime [min]	Type	Width [min]	Area [mAU*s]	Height [mAU]	Area %
1	4.311	BV	0.1077	69.33904	9.89981	0.3646
2	4.479	VV	0.1678	119.82873	10.60920	0.6301
3	4.621	VB	0.1634	107.53255	8.74918	0.5654
4	21.334	BB	0.5805	9498.18066	240.64174	49.9418
5	26.074	BB	0.6964	9223.63574	197.66888	48.4982

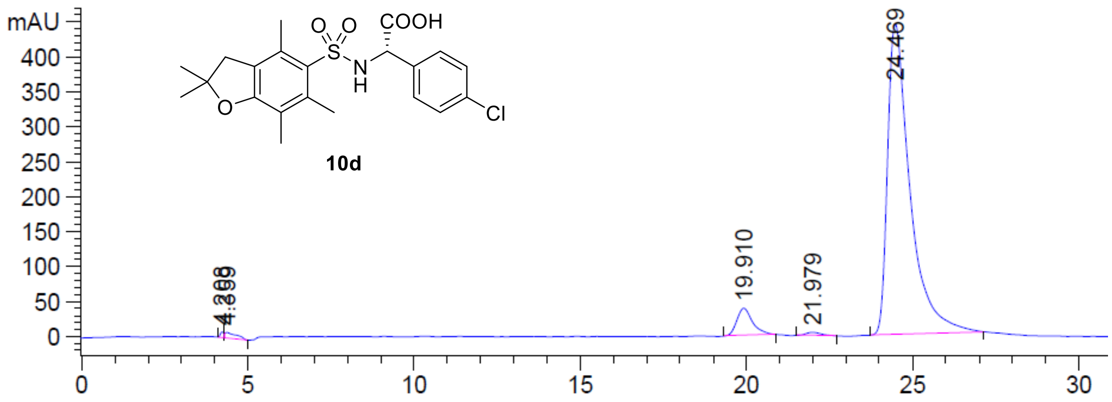

Signal 2: DAD1 B, Sig=254,16 Ref=380,100

Peak #	RetTime [min]	Type	Width [min]	Area [mAU*s]	Height [mAU]	Area %
1	4.208	BV	0.1227	62.44593	7.85594	0.2750
2	4.359	VV	0.3127	203.58652	8.05958	0.8965
3	19.910	BB	0.5085	1320.59668	39.25277	5.8151
4	21.979	BB	0.4526	163.75943	4.86591	0.7211
5	24.469	BB	0.6823	2.09594e4	444.35391	92.2924

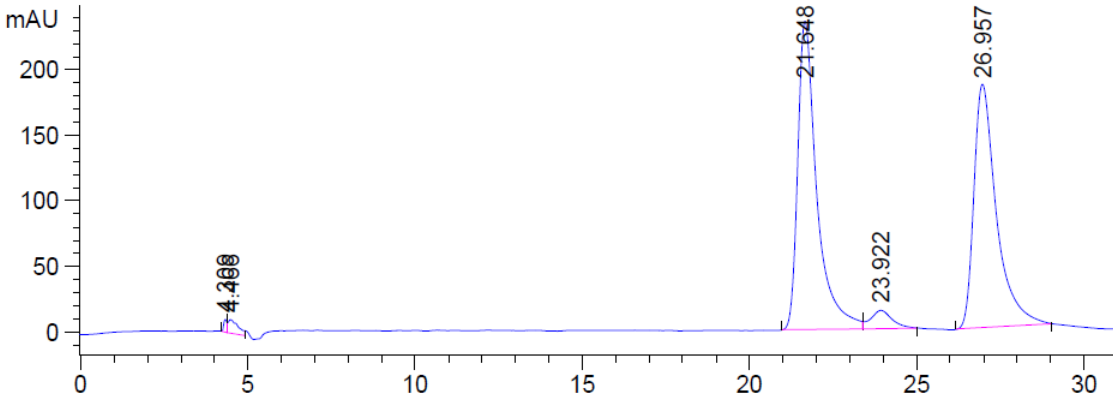

Signal 2: DAD1 B, Sig=254,16 Ref=380,100

Peak #	RetTime [min]	Type	Width [min]	Area [mAU*s]	Height [mAU]	Area %
1	4.309	BV	0.1083	68.42589	9.70096	0.3593
2	4.466	VB	0.2637	215.05797	10.29743	1.1294
3	21.648	BB	0.5898	9361.87988	235.47879	49.1641
4	23.922	BB	0.6231	633.47345	14.07428	3.3267
5	26.957	BB	0.7057	8763.26855	186.00394	46.0205

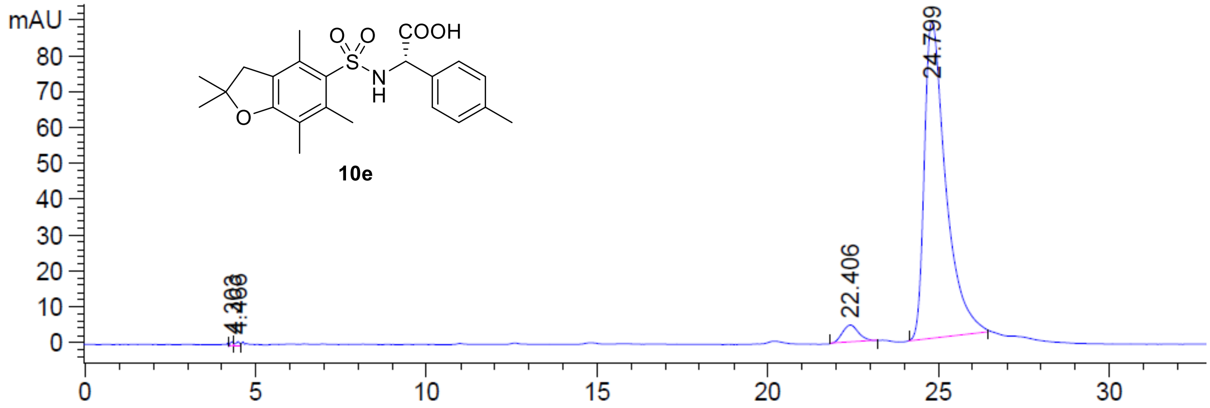

Signal 2: DAD1 B, Sig=254,16 Ref=380,100

Peak #	RetTime [min]	Type	Width [min]	Area [mAU*s]	Height [mAU]	Area %
1	4.232	BV	0.1646	118.84599	10.14943	0.4887
2	4.403	VV	0.1452	113.20203	10.89388	0.4655
3	4.532	VV	0.2362	165.12392	8.99320	0.6790
4	14.791	BB	0.2280	17.13867	1.06362	0.0705
5	20.199	BB	0.4332	199.94890	6.64255	0.8221
6	22.405	BB	0.5502	965.18524	26.54658	3.9686
7	24.798	MM	0.8031	2.27409e4	471.92319	93.5057

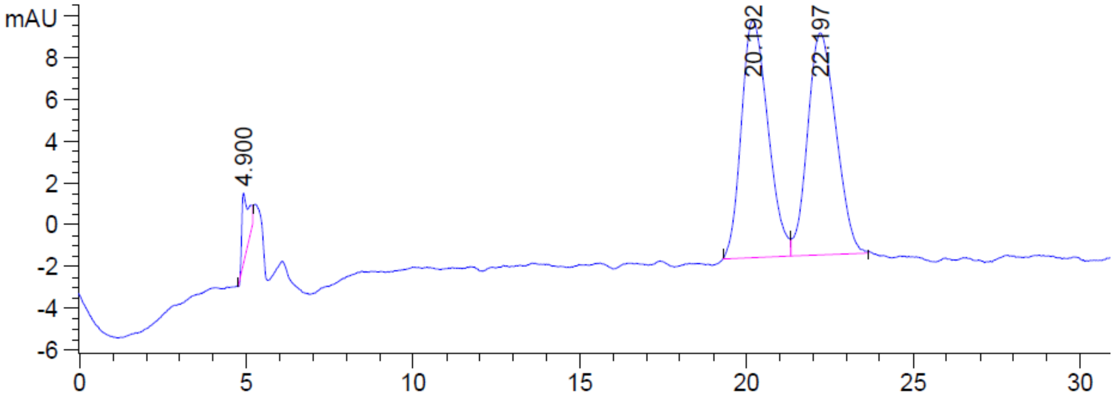

Signal 2: DAD1 B, Sig=254,16 Ref=380,100

Peak #	RetTime [min]	Type	Width [min]	Area [mAU*s]	Height [mAU]	Area %
1	4.900	BB	0.1789	44.67887	3.40936	3.4088
2	20.192	BV	0.6723	633.50110	11.36460	48.3327
3	22.197	VB	0.7197	632.53033	10.63719	48.2586

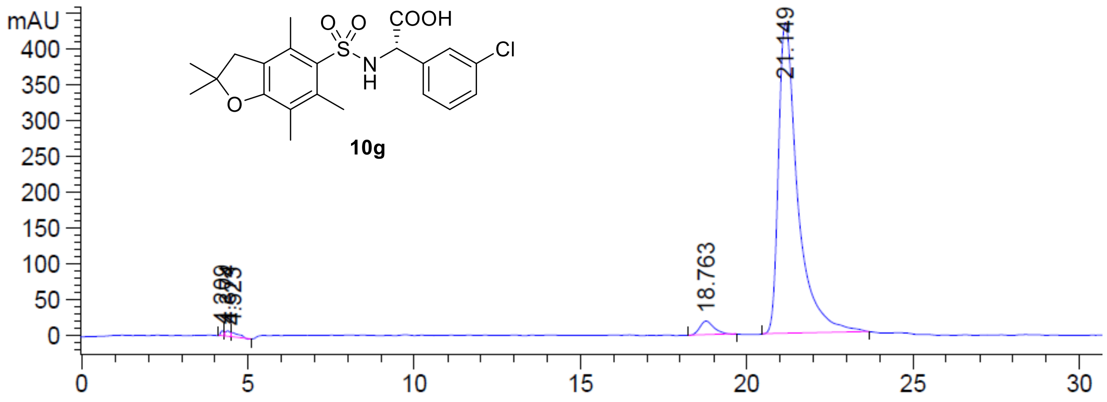

Signal 2: DAD1 B, Sig=254,16 Ref=380,100

Peak #	RetTime [min]	Type	Width [min]	Area [mAU*s]	Height [mAU]	Area %
1	4.209	BV	0.1244	57.63720	7.27653	0.3117
2	4.374	VV	0 1421	81.93240	8.09693	0.4431
3	4.523	VB	0.2099	103.18200	6.40591	0.5581
4	18.763	BB	0.4527	607.88788	19.66129	3.2878
5	21.149	BB	0.6005	1.76387e4	435.54517	95.3993

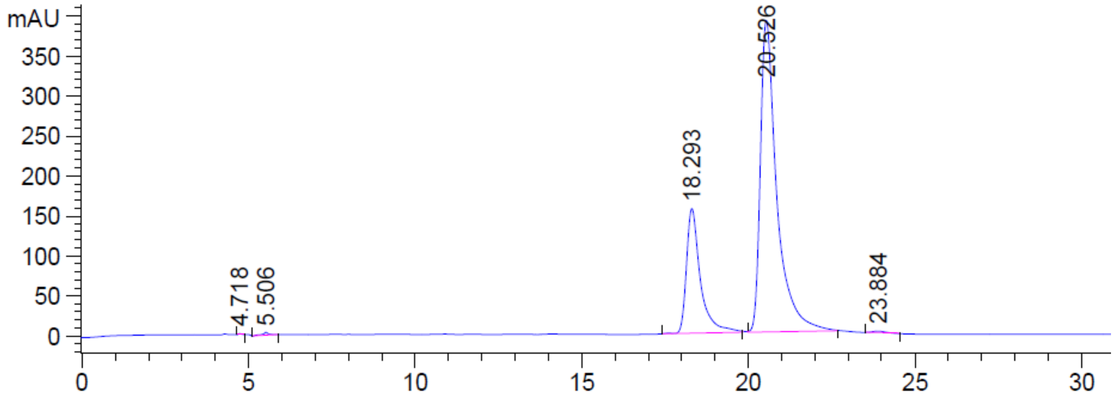

Signal 2: DAD1 B, Sig=254,16 Ref=380,100

Peak #	RetTime [min]	Type	Width [min]	Area [mAU*s]	Height [mAU]	Area %
1	4.718	BB	0.0927	9.25484	1.48470	0.0511
2	5.506	BB	0.2204	69.81905	4.10569	0.3853
3	18.293	BB	0.4310	4589.45166	156.08591	25.3302
4	20.526	BB	0.5071	1.33879e4	389.38815	73.8906
5	23.884	BB	0.3618	62.11916	2.07170	0.3428

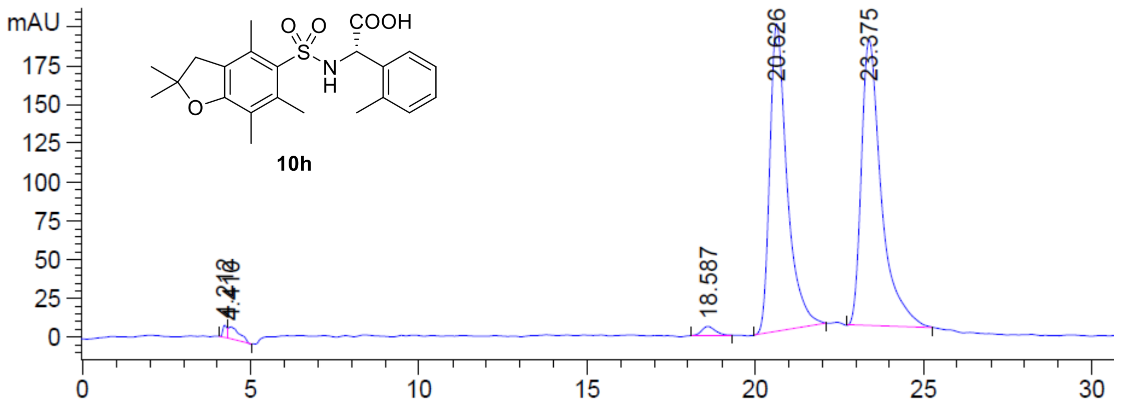

Signal 2: DAD1 B, Sig=254,16 Ref=380,100

Peak #	RetTime [min]	Type	Width [min]	Area [mAU*s]	Height [mAU]	Area %
1	4.212	BV	0.1383	74.45555	8.17406	0.4898
2	4.410	VB	0.2830	188.11340	8.09794	1.2375
3	18.587	BB	0.4087	161.91408	5.81760	1.0651
4	20.626	BB	0.5399	7134.26709	198.22079	46.9318
5	23.375	BB	0.6177	7642.60449	184.32230	50.2758

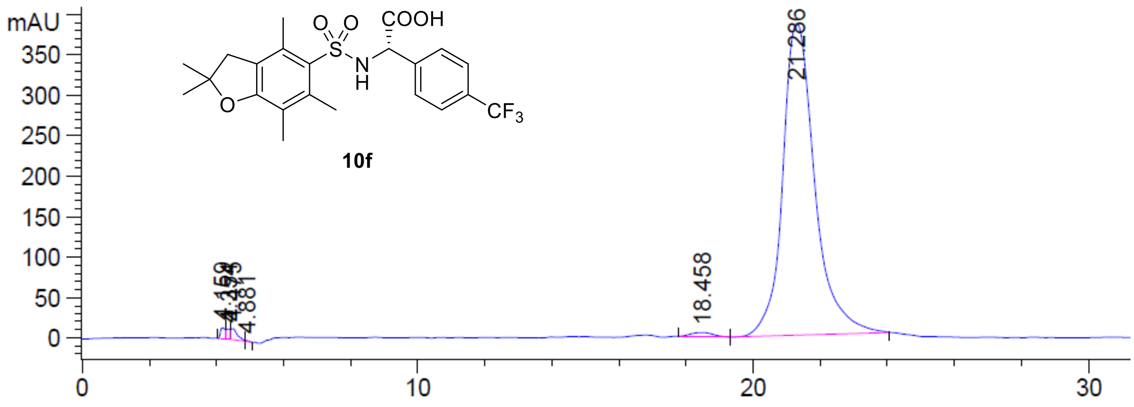

Signal 2: DAD1 B, Sig=254,16 Ref=380,100

Peak #	RetTime [min]	Type	Width [min]	Area [mAU*s]	Height [mAU]	Area %
1	4.159	BV	0.1515	140.75821	13.74558	0.5310
2	4.354	VV	0.1154	101.30814	13.21413	0.3822
3	4.475	VV	0.2041	201.56927	14.32734	0.7604
4	4.881	VV	0.0858	9.75013	1.50088	0.0368
5	18.458	BV	0.5898	284.43170	5.68395	1.0730
6	21.286	VB	1.0267	2.57491e4	385.75967	97.1407
7	41.724	BB	0.2556	20.08384	1.06715	0.0758

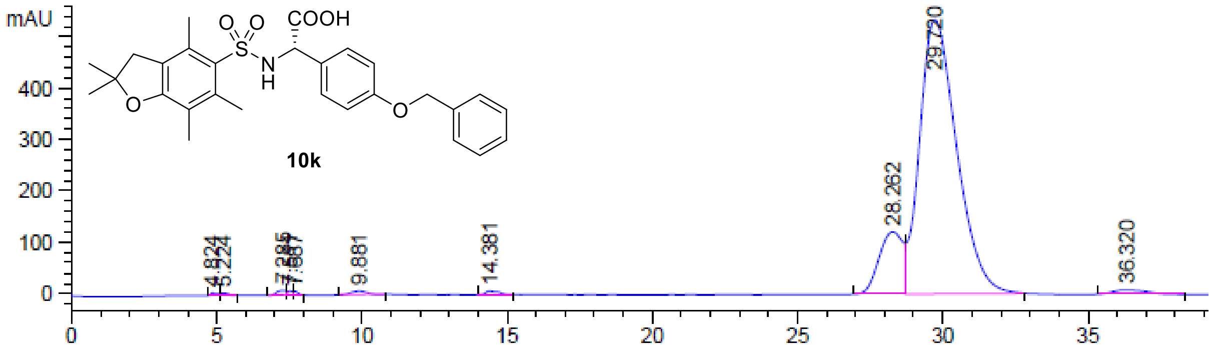

Signal 2: DAD1 B, Sig=254,16 Ref=380,100

Peak #	RetTime [min]	Type	Width [min]	Area [mAU*s]	Height [mAU]	Area %
1	4.824	BB	0.2130	88.30775	5.56865	0.1581
2	5.224	BB	0.2407	87.76944	4.64068	0.1571
3	7.285	BV	0.3117	217.32234	9.26257	0.3891
4	7.511	VV	0.1506	88.38148	8.40727	0.1582
5	7.687	VB	0.1715	83.58979	7.30491	0.1497
6	9.881	BB	0.4947	257.87762	7.11827	0.4617
7	14.381	BB	0.4149	219.94496	6.99290	0.3938
8	28.262	BV	0.8583	6773.98291	121.15233	12.1277
9	29.720	VB	1.3142	4.73185e4	532.78607	84.7158
10	36.320	BB	0.9930	719.92389	8.61431	1.2889

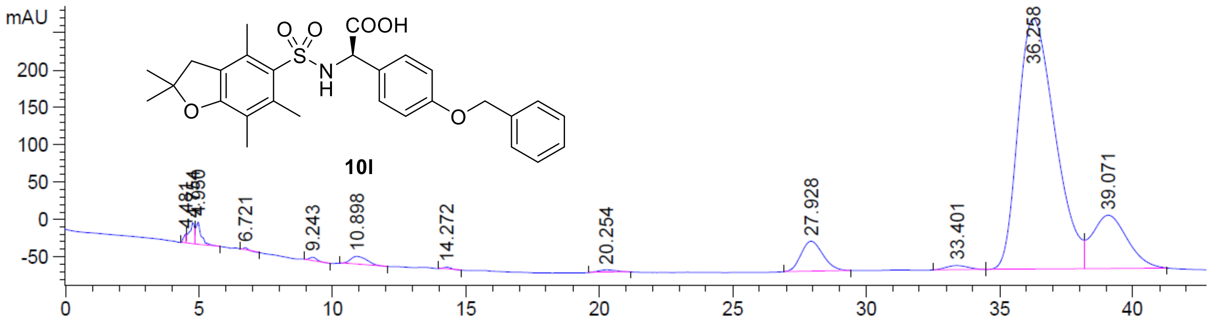

Signal 2: DAD1 B, Sig=254,16 Ref=380,100

Peak #	RetTime [min]	Type	Width [min]	Area [mAU*s]	Height [mAU]	Area %
1	4.481	BV	0.1040	89.46231	11.86282	0.1917
2	4.754	VV	0.1757	365.67953	28.12002	0.7836
3	4.950	VB	0.1855	416.09567	29.68783	0.8916
4	6.721	BB	0.1756	36.30733	3.12397	0.0778
5	9.243	BB	0.2927	83.51518	4.41555	0.1790
6	10.898	BB	0.5701	472.99051	10.77545	1.0135
7	14.272	BB	0.2958	54.66651	2.61616	0.1171
8	20.254	BB	0.6211	177.05392	3.45969	0.3794
9	27.928	BB	0.8343	2313.87622	40.27053	4.9581
10	33.401	BV	0.7178	339.25293	5.72094	0.7269
11	36.258	VV	1.4817	3.24442e4	335.64297	69.5205
12	39.071	VB	1.2795	6628.05469	71.76246	14.2024
13	48.092	BBA	1.1009	3247.39893	41.05018	6.9584

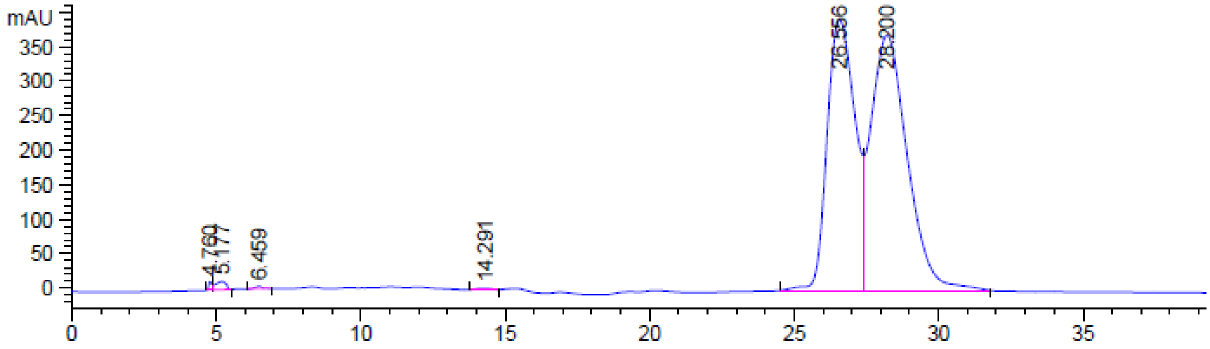

Peak #	RetTime [min]	Type	Width [min]	Area [mAU*s]	Height [mAU]	Area %
1	4.760	BV	0.1227	104.16186	12.31539	0.1747
2	5.177	VV	0.3398	295.68472	11.98631	0.4959
3	6.459	BB	0.2743	72.27960	3.66121	0.1212
4	14.291	BV	0.4050	66.03365	2.01140	0.1108
5	26.556	BV	1.0474	2.64803e4	394.34619	44.4150
6	28.200	VB	1.2837	3.26017e4	370.83386	54.6824

4 NMR data

^{1}H NMR 10a

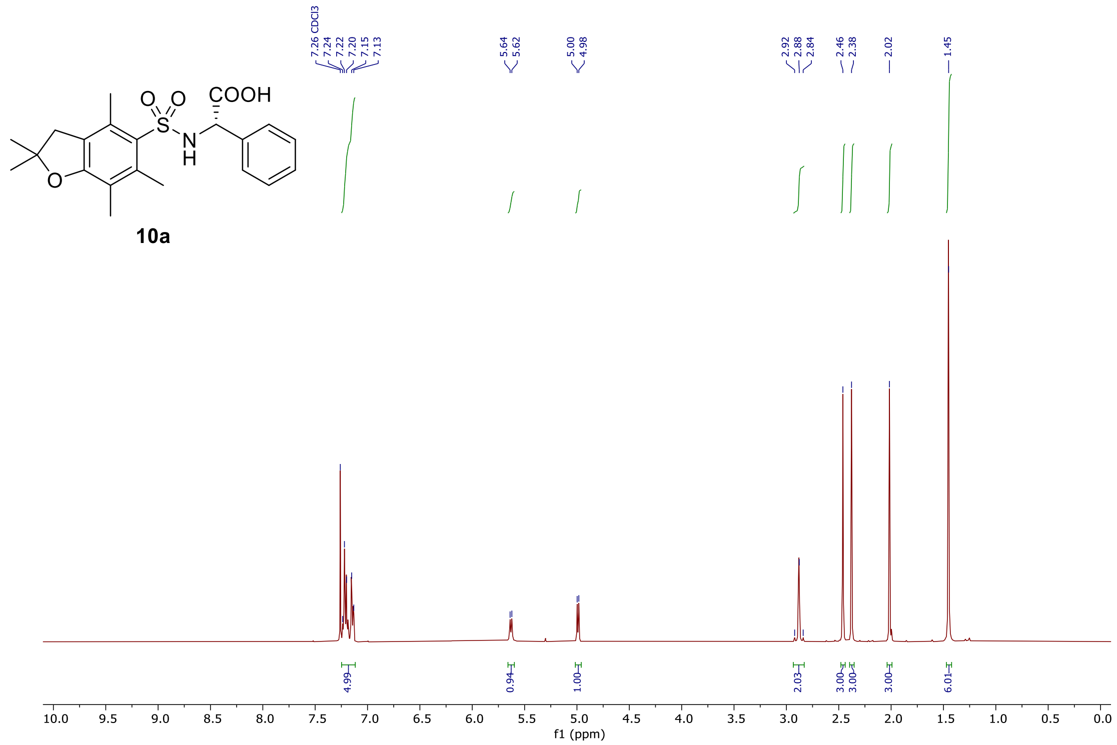

^{13}C NMR 10a

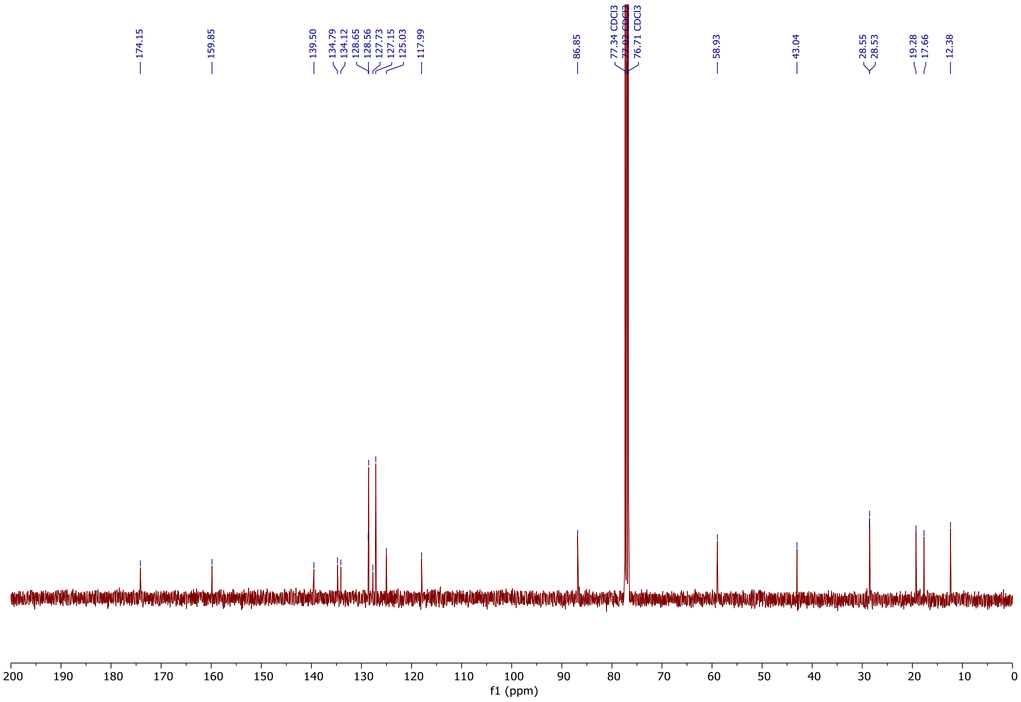

^{1}H NMR 10b

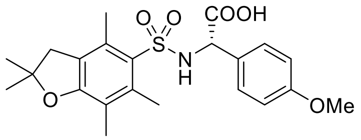

10b

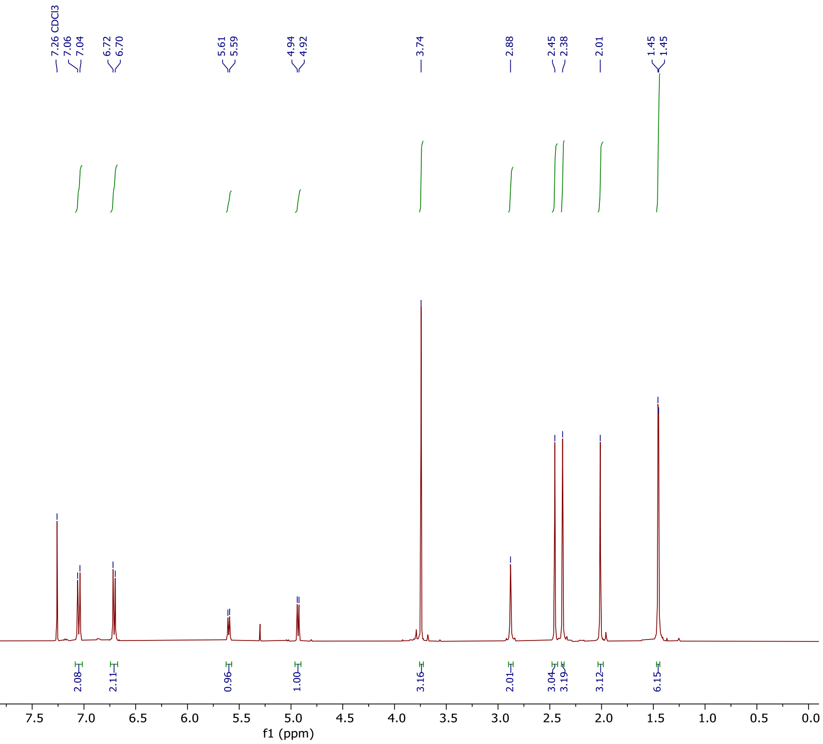

^{13}C NMR 10b

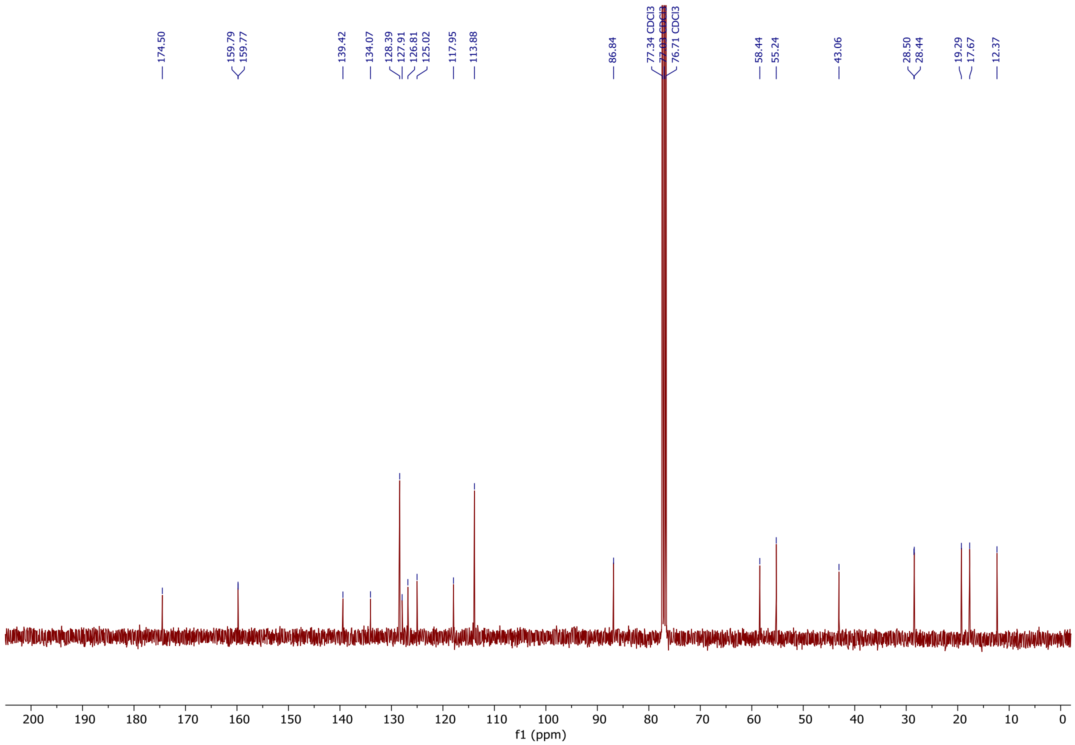

^{1}H NMR 10c

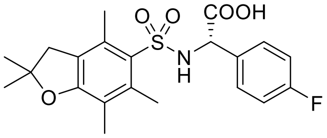

10c

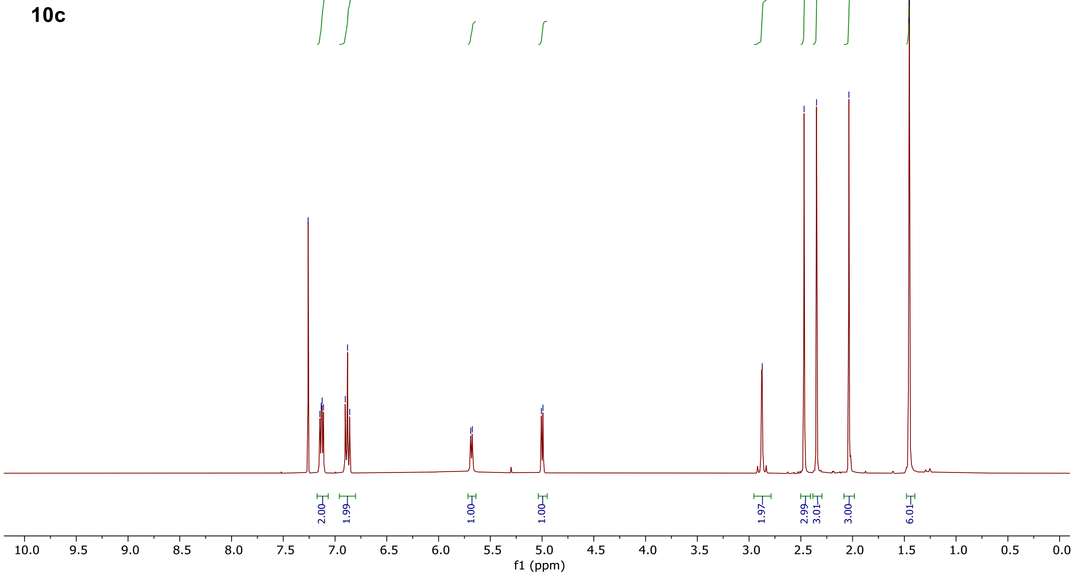

^{13}C NMR 10c

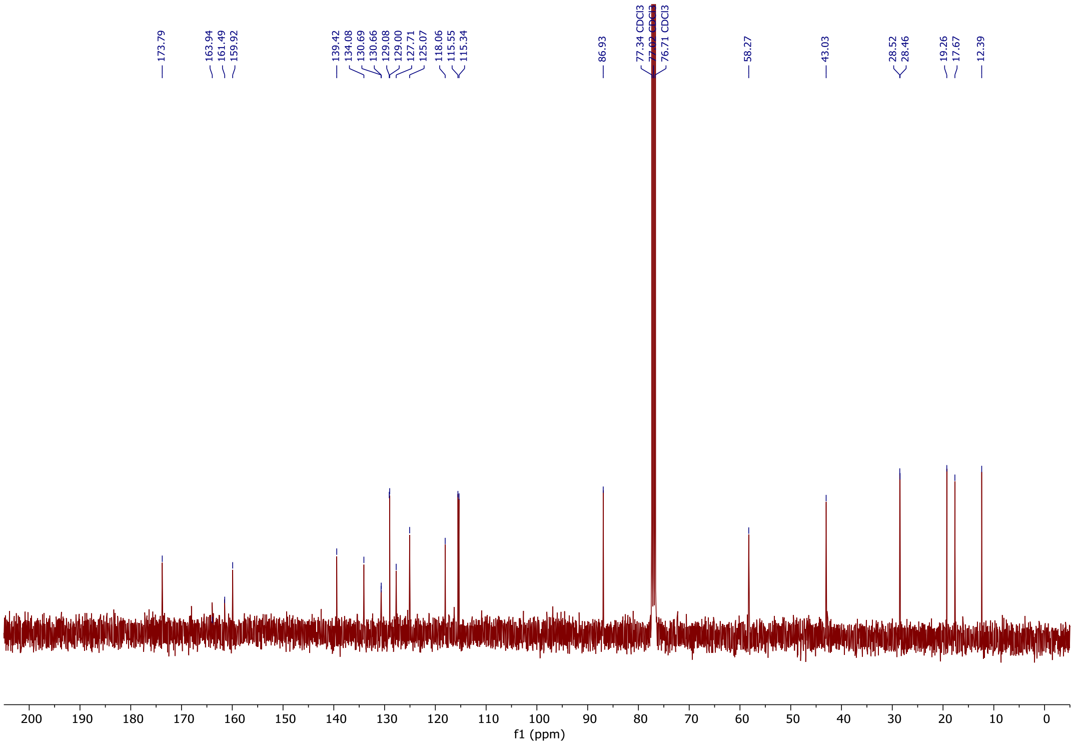

^{19}F NMR 10c

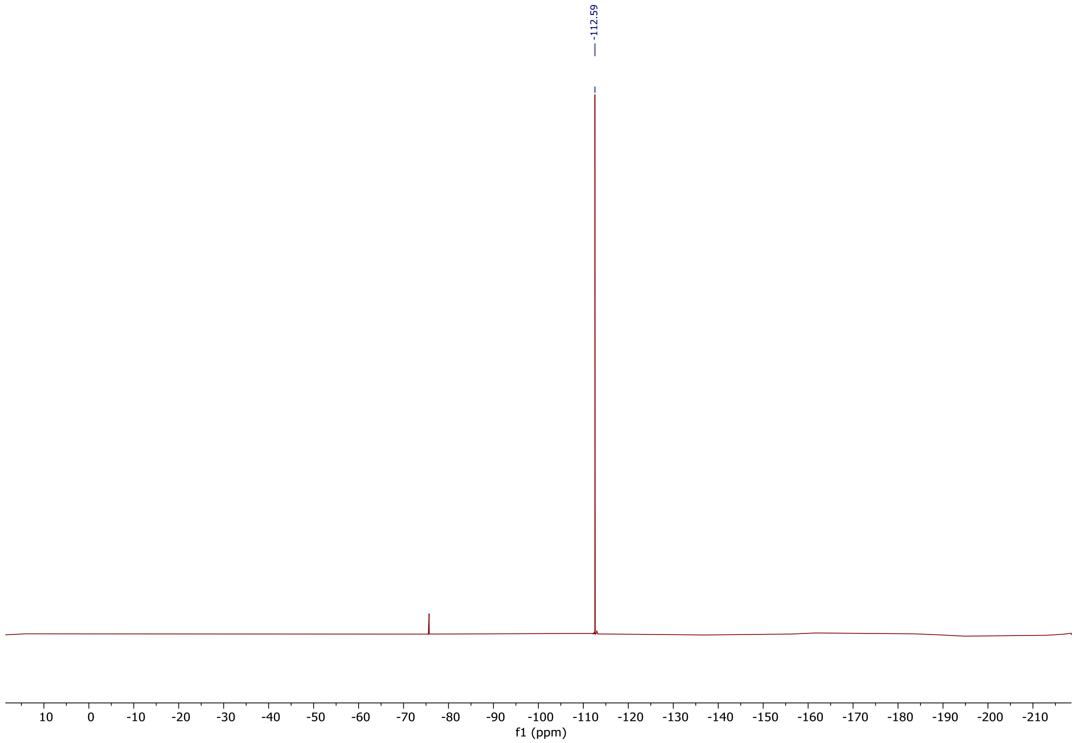

^{1}H NMR 10d

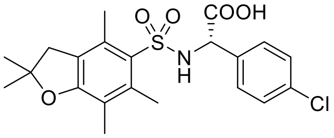

10d

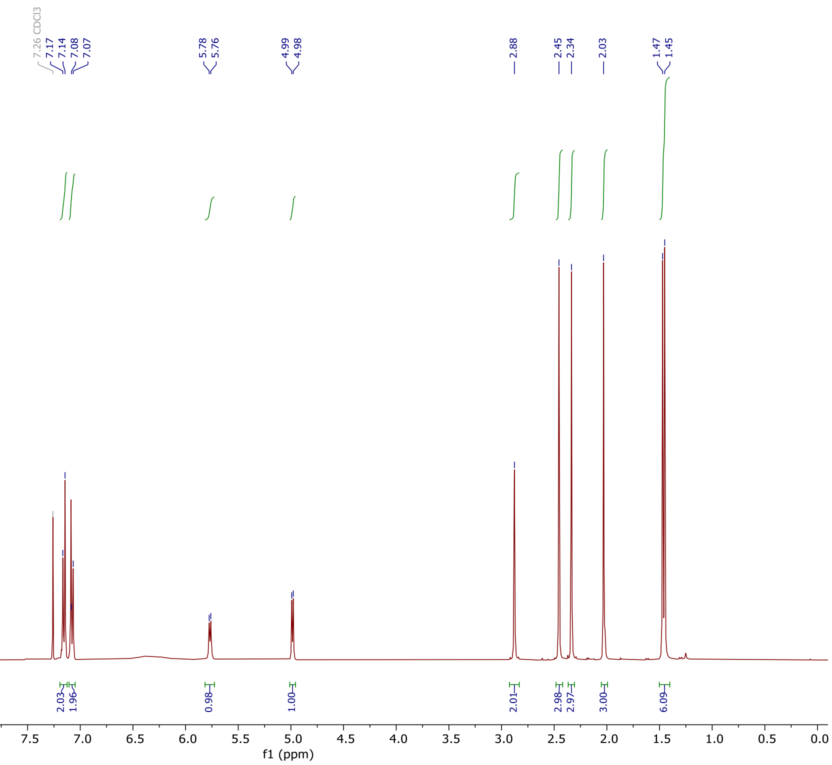

^{13}C NMR 10d

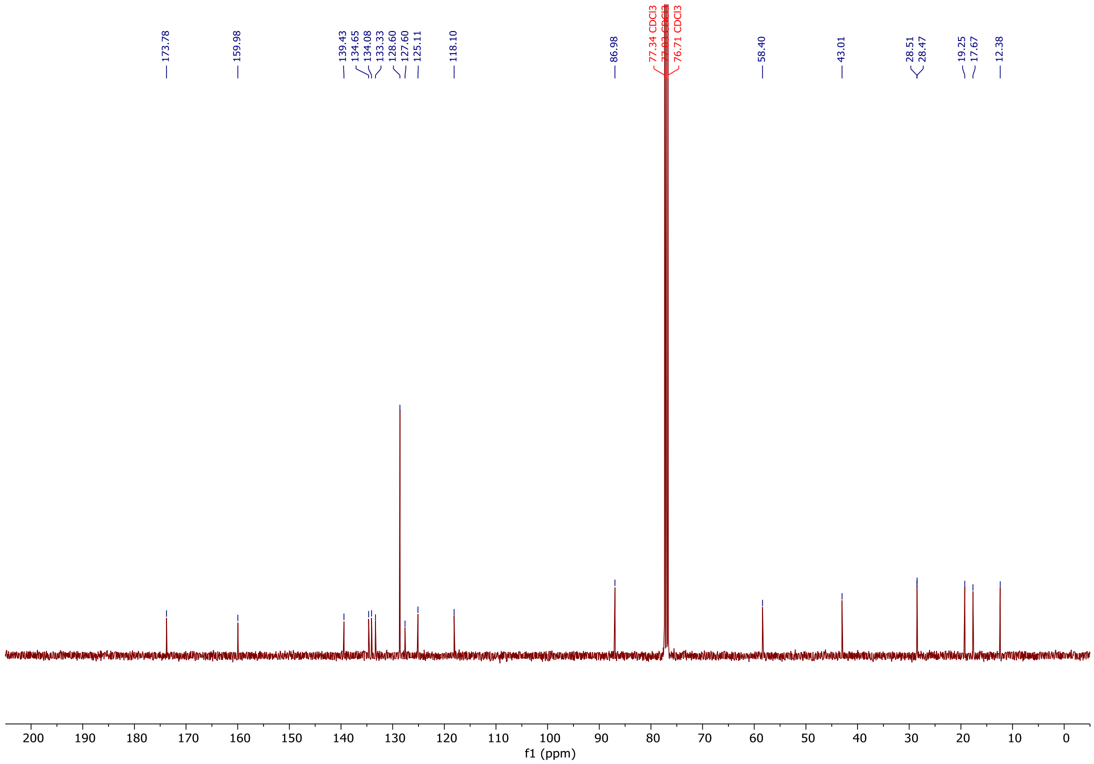

^{1}H NMR 10e

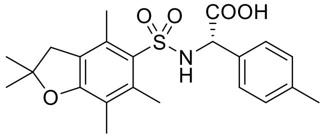

10e

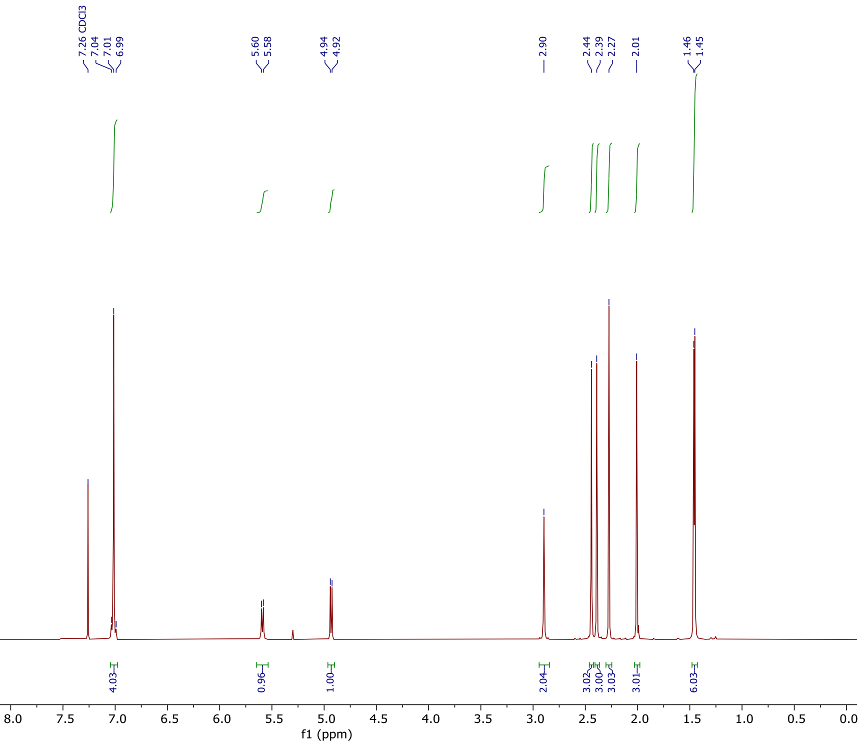

^{13}C NMR 10e

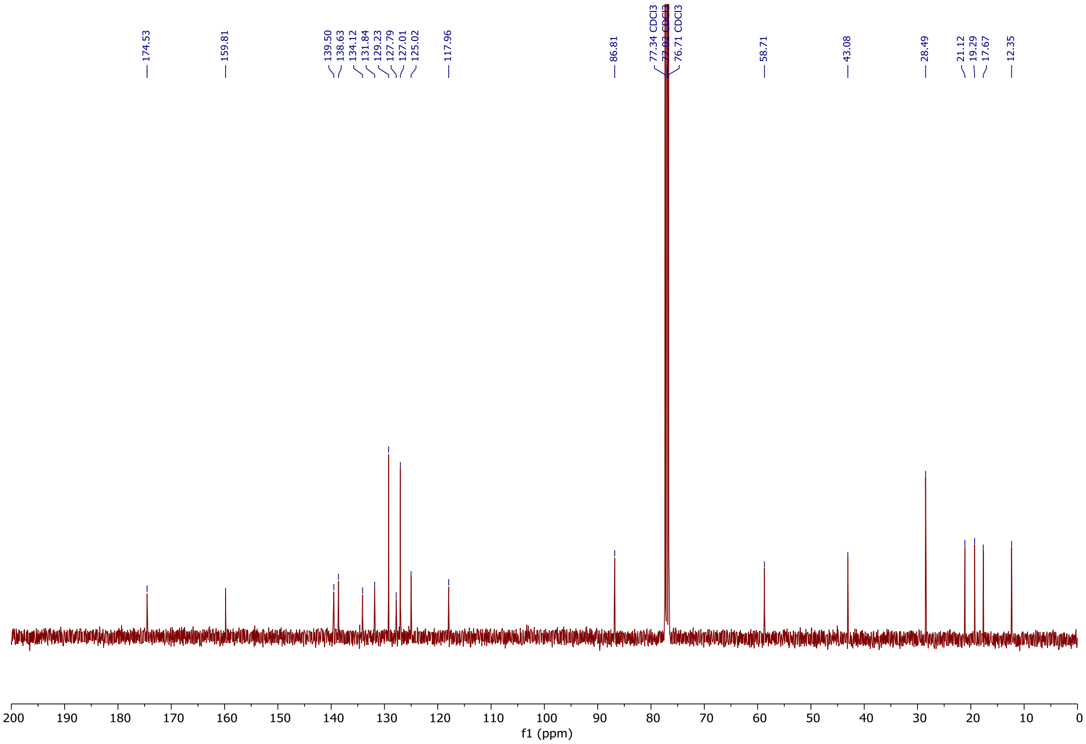

^{1}H NMR 10f

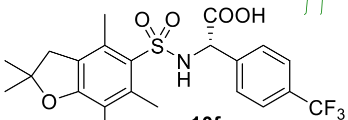

10f

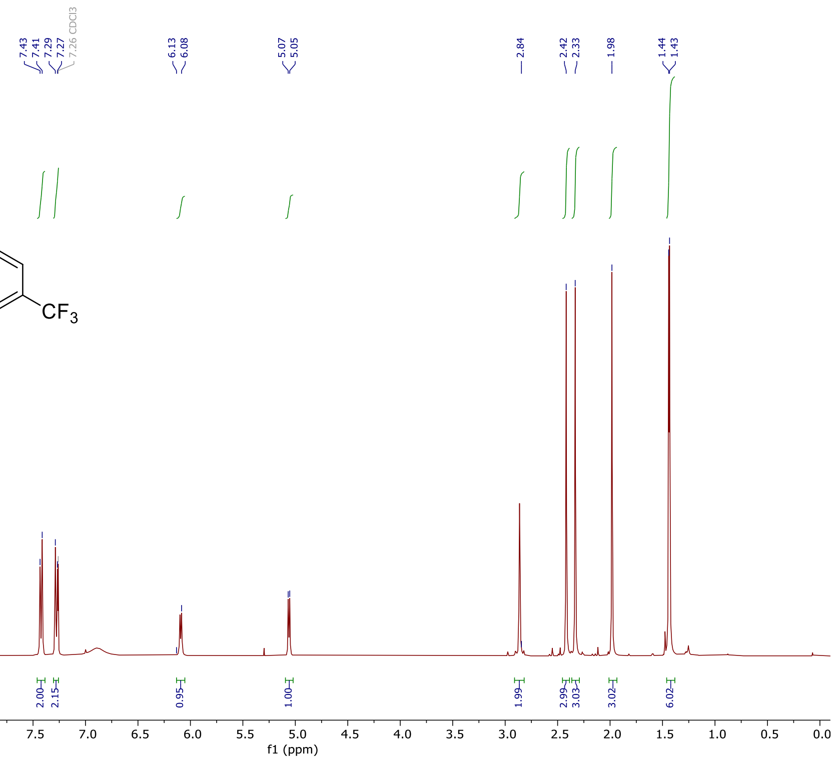

^{13}C NMR 10f

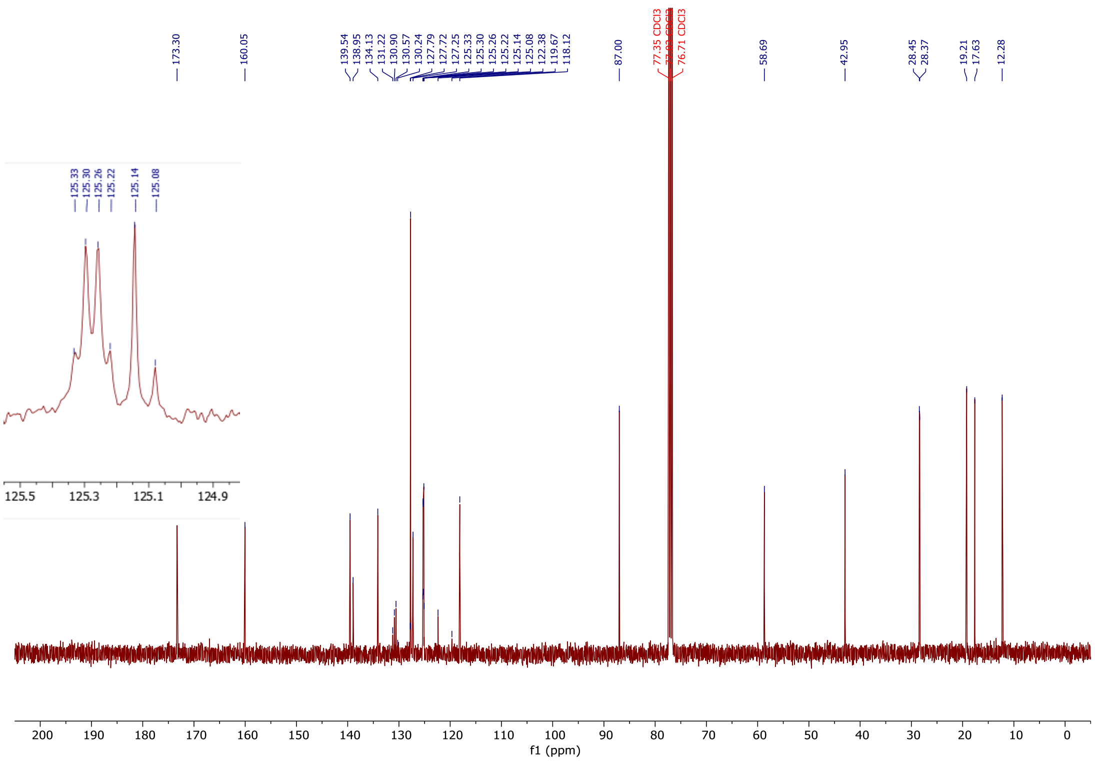

^{19}F NMR 10f

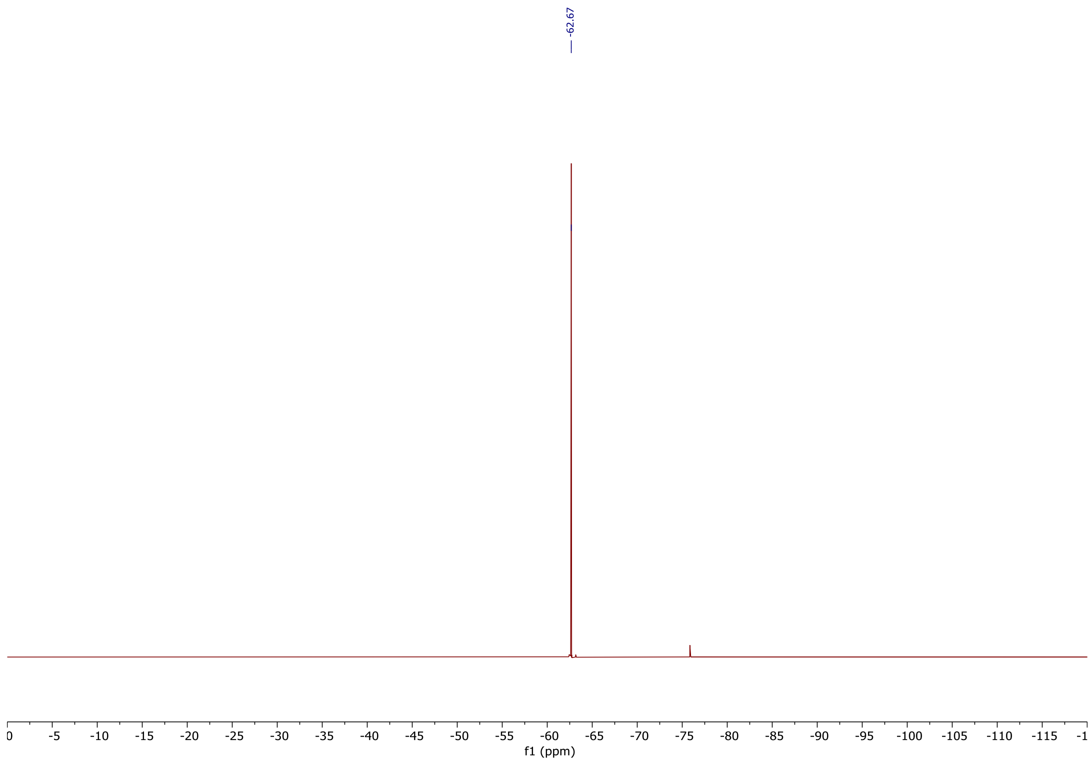

^{1}H NMR 10g

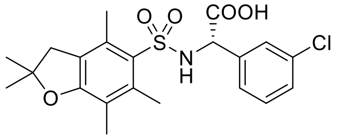

10g

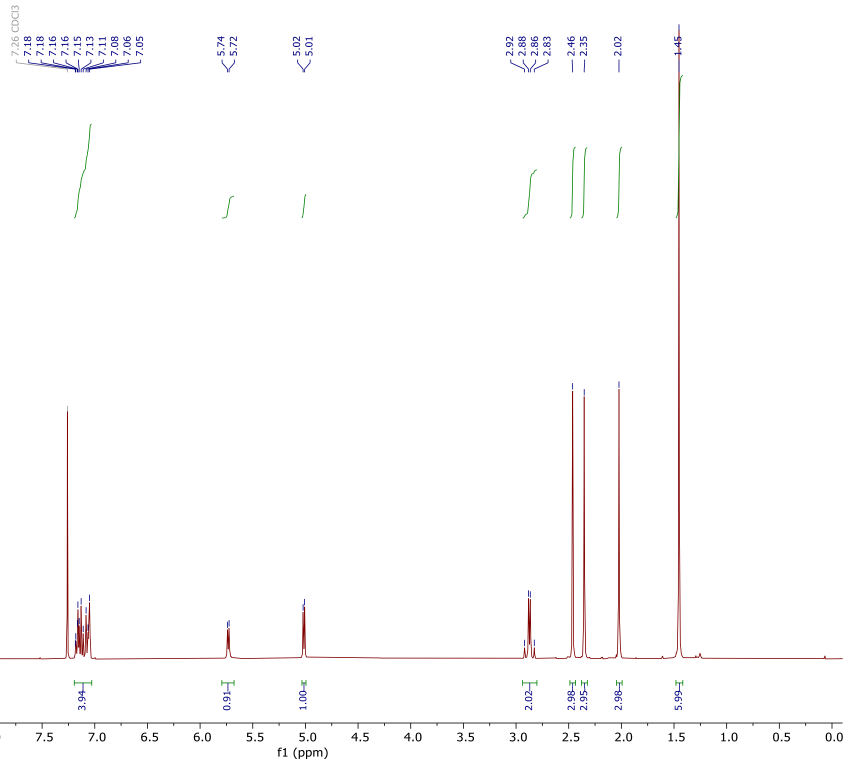

^{13}C NMR 10g

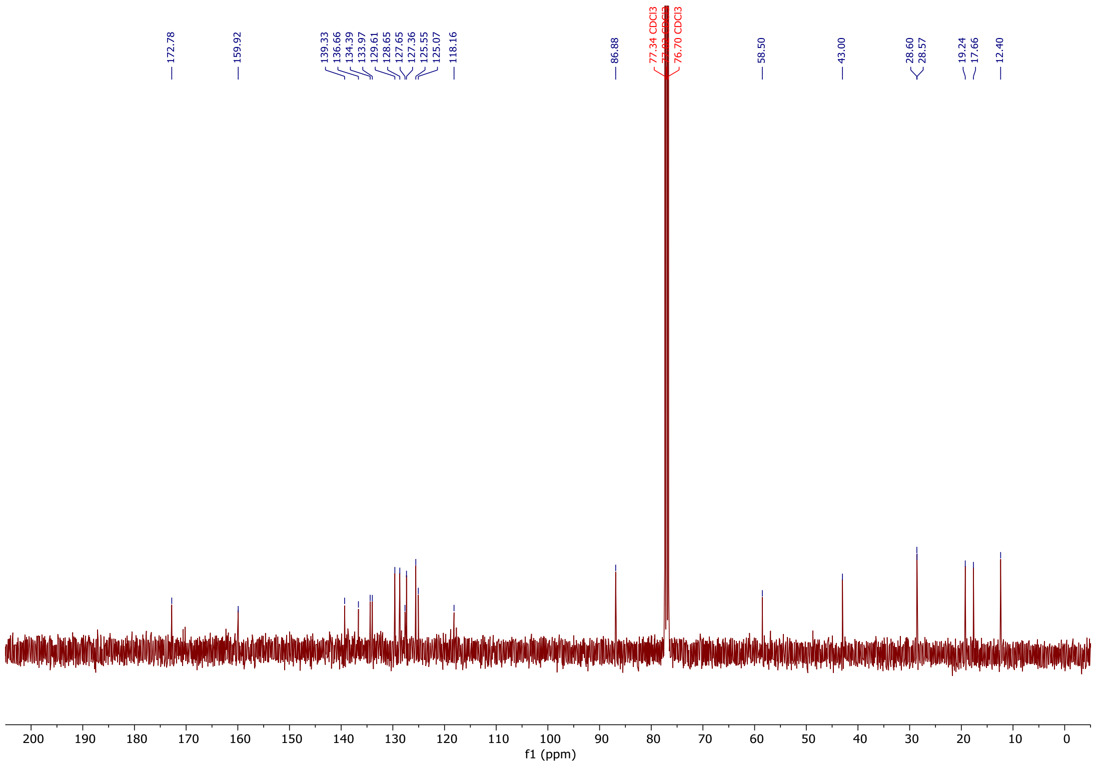

^{1}H NMR 10h

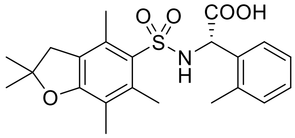

10h

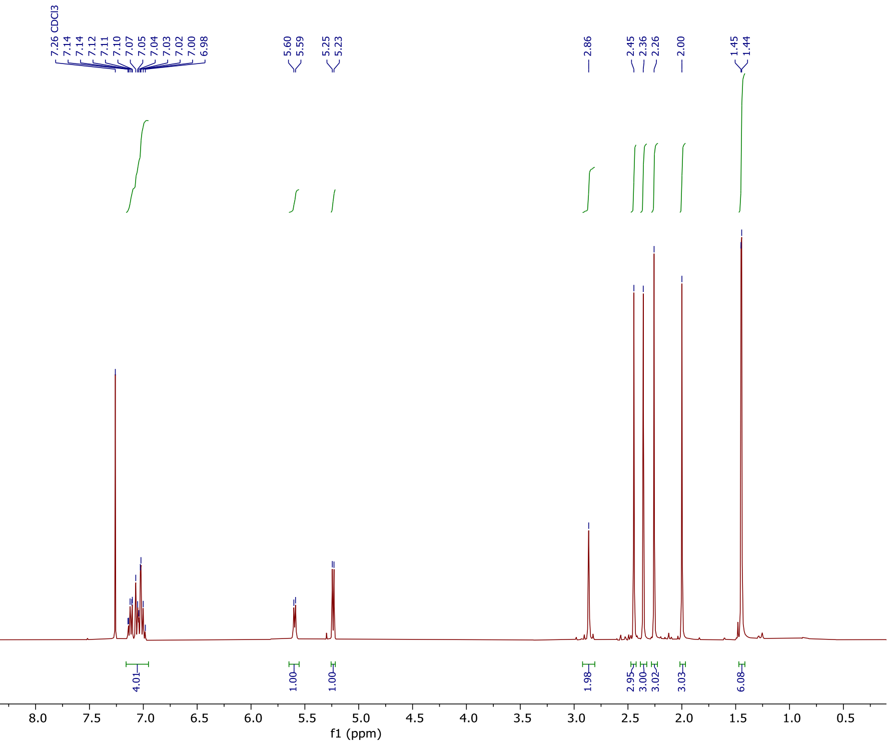

^{13}C NMR 10h

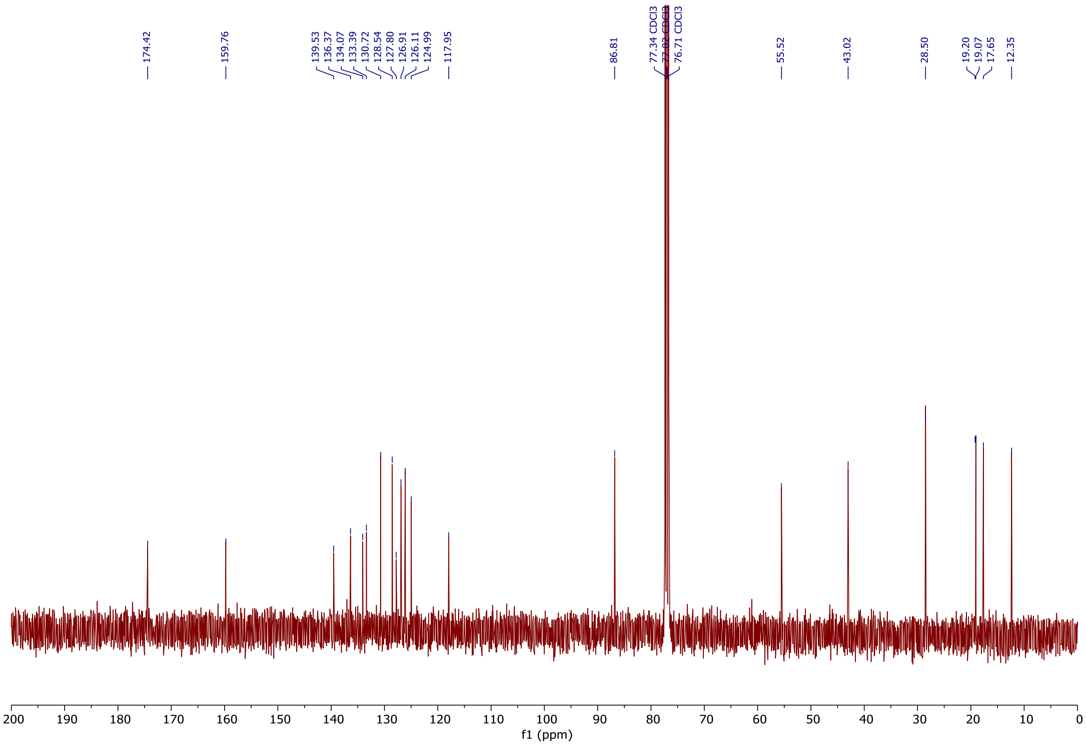

^{1}H NMR 10k

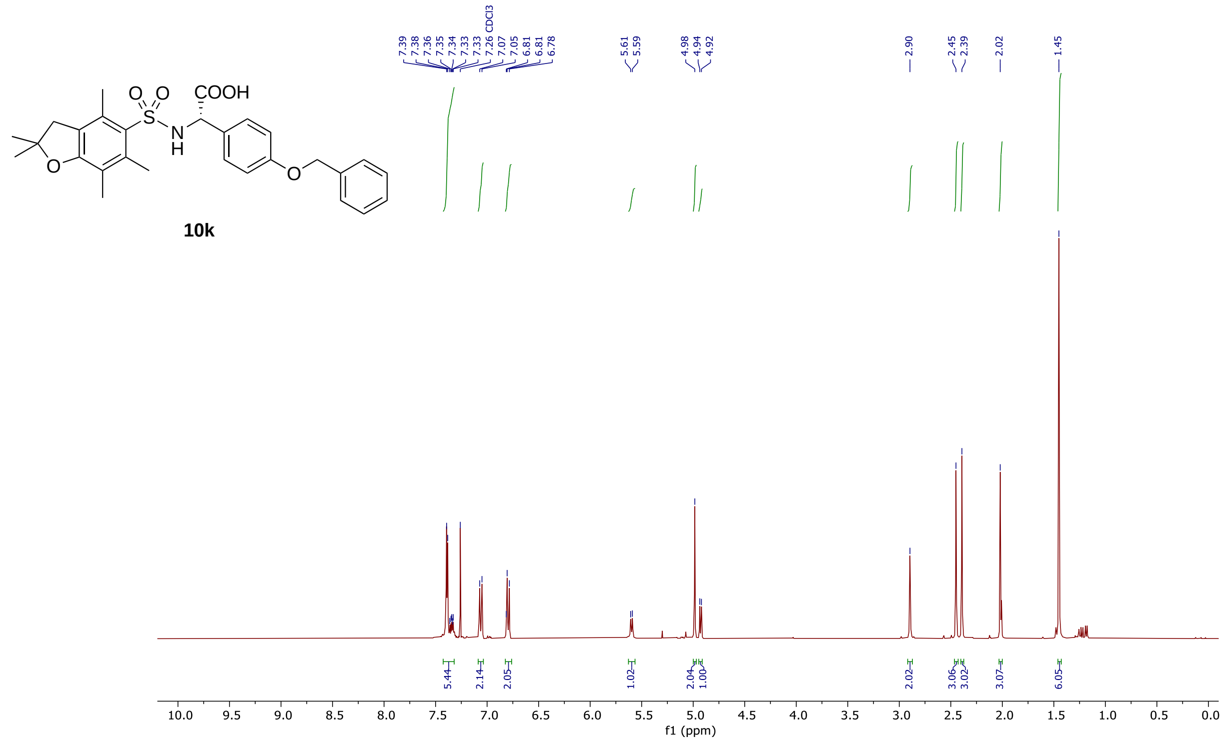

^{13}C NMR 10k

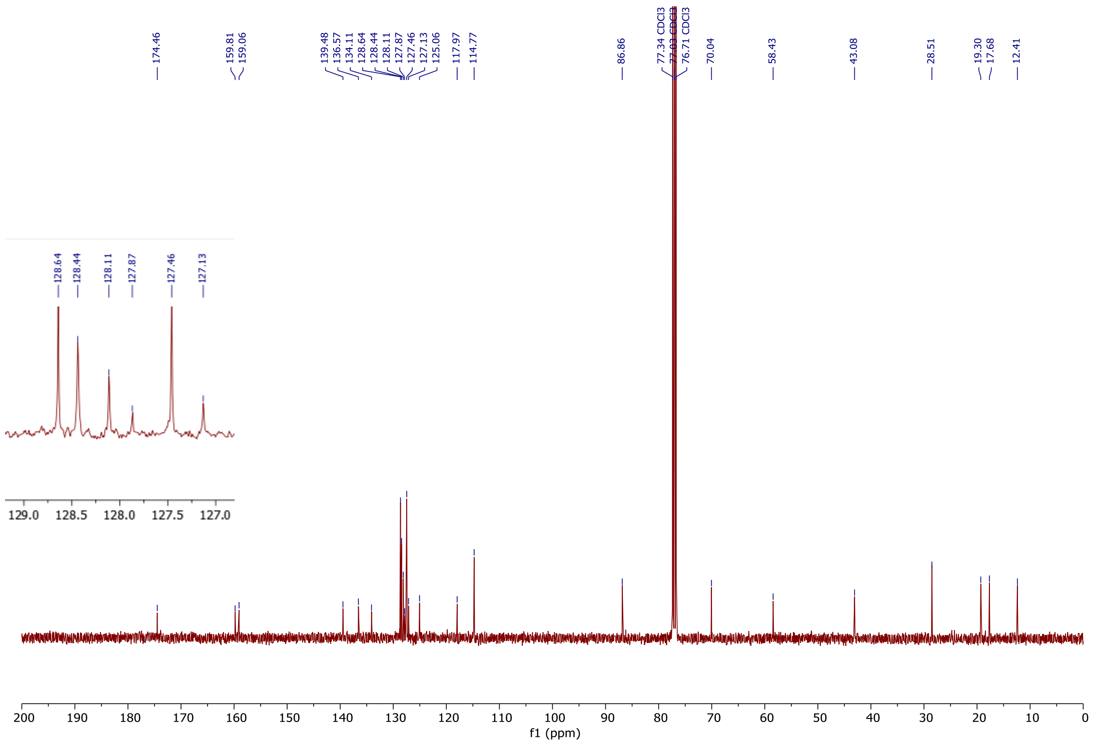

^{1}H NMR 10l

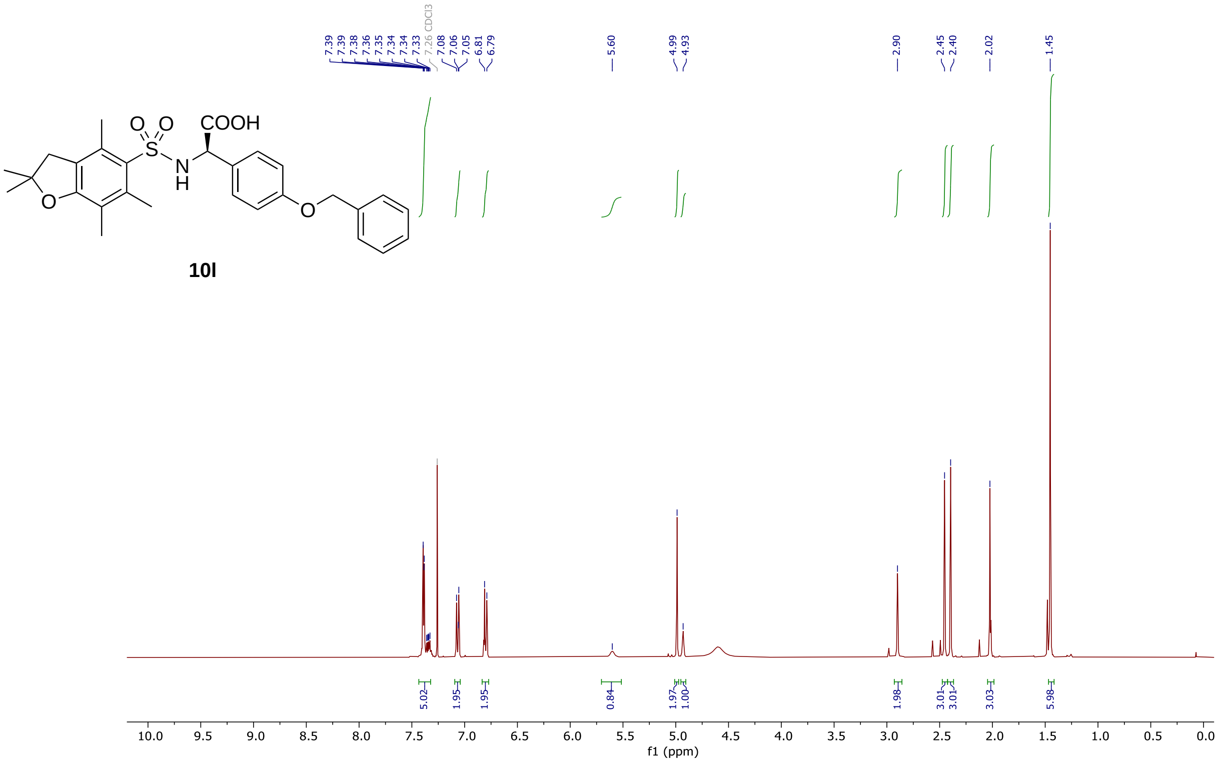

10l

5 References

[1] Carpino, L. A., Shroff, H., Triolo, S. A., Mansour, E.-S. M. E., Wenschuh, H. and Albericio, F. *Tetrahedron Letters*, **1993**, *34*, 7829-7832. https://doi.org/10.1016/S0040-4039(00)61487-9

[2] Denmark, S. E., Stavenger, R. A., Faucher, A.-M., and Edwards, J. P. *The Journal of Organic Chemistry*, **1997,** *62*, 3375-3389. https://doi.org/10.1021/jo970044z

[3] Lennox, A. J. J. *Organotrifluoroborate Preparation. In: Organotrifluoroborate Preparation, Coupling and Hydrolysis*, **2013**, Springer Theses, Springer International Publishing Switzerland. https://doi.org/10.1007/978-3-319-01134-9_2

[4] Jakob, B., Diehl, A.M., Horst, K., Kelm, H. and Manolikakes, G. *Frontiers in Chemistry*, **2023**, 11:1165618. https://doi.org/10.3389/fchem.2023.1165618
